# Superior Dislocation of the Mandibular Condyle into the Middle Cranial Fossa: A Comprehensive Review of the Literature

**DOI:** 10.3390/jcm12113781

**Published:** 2023-05-31

**Authors:** Kazuya Yoshida

**Affiliations:** Department of Oral and Maxillofacial Surgery, National Hospital Organization, Kyoto Medical Center, 1-1 Mukaihata-cho, Fukakusa, Fushimi-ku, Kyoto 612-8555, Japan; yoshida.kazuya.ut@mail.hosp.go.jp; Tel.: +81-75-641-9161

**Keywords:** mandibular condyle, superior dislocation, middle cranial fossa, glenoid fossa, fracture, intrusion, reduction, craniotomy

## Abstract

The superior dislocation of the condyle into the cranium occasionally requires invasive procedures due to the absence of a timely diagnosis. This review analyzed the available clinical data to provide information on treatment decisions. The reports were assessed using electronic medical databases from inception to 31 October 2022. A total of 116 cases from 104 studies were assessed; among the patients, 60% and 87.5% of the affected women and men required open reduction, respectively. The ratio of closed to open procedures within 7 days after injury was maintained; however, closed reduction decreased over time, and all cases required open reduction after 22 days. Eighty percent of the patients with a total intrusion of the condyle required open reduction, whereas the frequency for both procedures was comparable in the remaining patients. Open reduction was significantly more frequently performed for men (*p* = 0.026, odds ratio; 4.959, 95% confidence interval; 1.208–20.365) and less frequently performed in cases with partial intrusion (*p* = 0.011; odds ratio: 0.186; 95% confidence interval: 0.051–0.684); the frequency varied according to the time until treatment (*p* = 0.027, odds ratio; 1.124, 95% confidence interval; 1.013–1.246). Appropriate diagnostic imaging and prompt diagnosis are indispensable for minimally invasive treatment of this condition.

## 1. Introduction

Anatomical characteristics of the craniofacial structures generally prevent the cranium from being penetrated, including cases in which a strong superiorly and posteriorly directed force from blunt trauma to the chin is transmitted to the mandibular condyle. Therefore, penetration of the condyle through the glenoid fossa due to superior or central displacement is rare. In 1834, Lefèvre de Rochefort reported the first case of central dislocation of the mandibular condyle into the middle cranial fossa [1]. An intoxicated 22-year-old man fell on his chin from a second-floor window. He was completely unable to open his mouth; his maxilla and mandible were in contact, with the mandible slightly deviated posteriorly and laterally. After the injury, he developed a severe headache and died of convulsions six months later. A large brain abscess and intracranial penetration of the right mandibular condyle were confirmed with an autopsy [1]. Although this type of condylar dislocation is extremely rare due to anatomical and biomechanical factors, numerous authors have since reported this entity in case reports or case series [1,2,3,4,5,6,7,8,9,10,11,12,13,14,15,16,17,18,19,20,21,22,23,24,25,26,27,28,29,30,31,32,33,34,35,36,37,38,39,40,41,42,43,44,45,46,47,48,49,50,51,52,53,54,55,56,57,58,59,60,61,62,63,64,65,66,67,68,69,70,71,72,73,74,75,76,77,78,79,80,81,82,83,84,85,86,87,88,89,90,91,92,93,94,95,96,97,98,99,100,101,102,103,104,105,106,107,108,109].

Dislocation of the temporomandibular joint (TMJ) is a relatively common event, with an estimated incidence of 2.5–25 per 100,000 individuals per year [110,111]. Dislocation of the TMJ refers to the displacement of the head of the mandibular condyle from its normal position in the glenoid fossa. The dislocation can be anterior, posterior, medial, lateral, or superior. Anterior dislocations are most frequently observed, whereas dislocations in the posterior, medial, or lateral direction are less common. A systematic review of a TMJ dislocation reported 79 acute, 35 chronic, and 311 recurrent cases [112]. Among the 425 cases, a superior dislocation was observed in only 8 cases [112]. Mandibular condyle fractures account for 37.4% [113] or 34% [114] of all mandibular fractures. However, an intracranial displacement of the mandibular condyle is rare because of the safety mechanism that protects the cranium and brain from injury caused by the penetration of the condyle [14]. 

Numerous authors have reviewed the central dislocation of the mandibular condyle into the middle cranial fossa [11,27,40,49,50,55,61,64,65,85,87,88,97,102,106,115,116]. However, most reviews are limited to reports published in English, although many important reports in the literature are written in languages other than English. This type of fracture has often been misdiagnosed as an anterior dislocation of the condyle, condylar neck fracture, or anterior disk displacement without reduction. In cases in which a closed reduction is possible in an early diagnosis, open surgical procedures, such as a craniotomy or osteotomy, may nevertheless be required in a delayed diagnosis. For such rare conditions, collecting data on as many cases as possible is necessary to decide whether a closed or open treatment is the optimal strategy. Therefore, this review comprehensively assessed reports on the dislocation of the mandibular condyle into the middle cranial fossa accessible in electronic databases without any language restrictions. This study aimed to analyze the clinical data and the factors that can be used by practitioners to decide between a closed and open reduction.

## 2. Materials and Methods

### 2.1. Review of Literature

**Figure 1:**
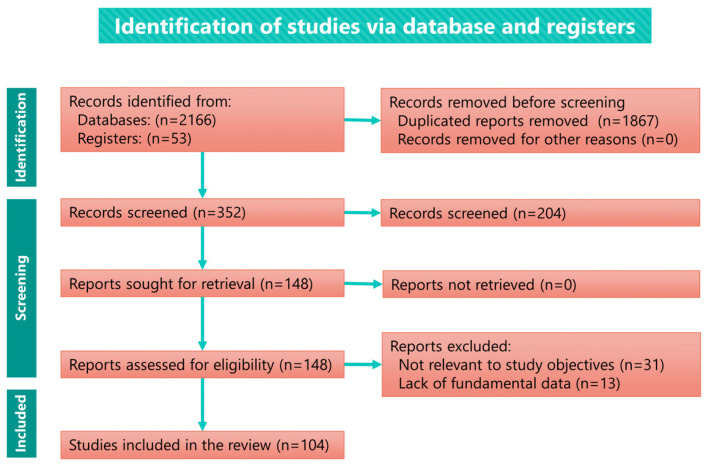
Diagram of the literature search and screening strategy.

The literature search strategy was based on comprehensive electronic medical literature databases (PubMed, Scopus, Web of Science, EBSCO, Ovid, Google Scholar, Japan Medical Abstracts Society, and Medical Online) using the keywords (“superior” OR “central” OR “intracranial” OR “traumatic”) AND (“dislocation” OR “penetration” OR “intrusion” OR “displacement” OR “luxation” OR “impaction” OR “migration” OR “fracture” OR “injury”) AND (“condyle” OR “mandibular condyle” OR “mandible” OR “temporomandibular joint”) AND (“middle cranial fossa” OR “cranial fossa” OR “glenoid fossa” OR “skull” OR ”brain”. Furthermore, a manual search was conducted for articles cited in related resources. Reports from 1834 to 31 October 2022 identified in these databases or via a manual search with no language restrictions were screened by the author [117]. Duplicate reports of the same case were excluded. The exclusion criteria were records with missing fundamental information, such as sex, age, etiology, or clinical presentation, and those irrelevant to the purpose of the study. All reports were assessed for eligibility and reviewed by the author (Figure 1). 

### 2.2. Analysis

Fundamental clinical data, including age, sex, affected side, etiology, chief complaint, diagnostic imaging, method of diagnosis, degree of intrusion, delay to diagnosis, maximal mouth opening, deviation, open bite, neurological symptoms (Table S1), treatment, and follow-up (Table S2), were extracted and evaluated. 

**Figure 2:**
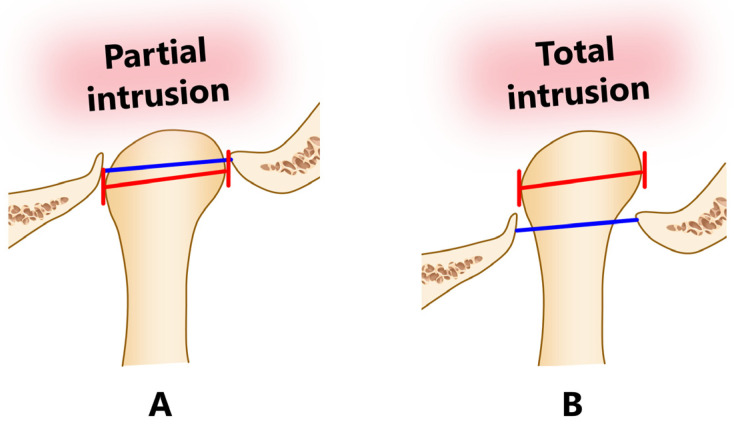
Degree of penetration of the condyle and glenoid fossa. Partial intrusion is characterized by the maximally bulging parts of the dislocated mandibular condyle (medial and lateral poles, red lines) not extending beyond the base of the skull (blue lines) (**A**), and total intrusion is characterized by the same parts extending beyond the aforementioned limit (complete penetration, **B**).

The degree of penetration of the condyle was divided into two categories, partial or total intrusion, based on the diagnostic imaging and the findings reported in the text of each article. Partial intrusion was defined as cases in which the maximally bulging parts of the dislocated mandibular condyle (medial and lateral poles; red lines in Figure 2) did not extend beyond the base of the skull (blue lines in Figure 2), and total intrusion was defined as complete penetration beyond the base of the skull.

### 2.3. Statistics

Age, sex, degree of penetration, and delay in treatment were analyzed using binomial logistic regression analysis to determine potential indicators that can be used to select treatments (closed vs. open procedure). The Fisher’s exact test was performed to assess the statistical significance of differences in distributions. A two-tailed paired *t*-test was used to evaluate changes before and after treatment. Differences in clinical data between the groups were compared using a one-way analysis of variance. The Bonferroni method was used to conduct a post hoc test when the analysis of variance revealed significant differences. All analyses were performed using the SPSS statistical software package for Windows (version 24.0; SPSS Japan, Inc. Tokyo, Japan). The null hypothesis was rejected at the 5% significance level (*p* < 0.05). 

## 3. Results

The number of reports retrieved from the literature search, assessed for eligibility and included in the comprehensivereview, are presented in the flow diagram (Figure 1). The total number of records retrieved from the databases was 2166 (PubMed, 303; Scopus, 360; Web of Science, 197; EBSCO, 1060; Google Scholar, 206; Japan Medical Abstracts Society, 20; and Medical Online, 20). Fifty-three records were additionally retrieved by manually searching relevant papers or books. The search yielded a total of 104 articles. The number of evaluated studies discriminated by their original language were as follows: English, 81; Japanese, 6; German 5; French, 5; Danish, 2; Italian, 2; Dutch, 1; Persian, 1; and Ukrainian, 1. The number of reports by the number of cases were as follows: 1 case, 94; 2 cases, 8; and 3 cases, 2. All studies were case reports or case series. Table S1 summarizes the demographic data and symptoms of the patients included in the reviewed reports. 

### 3.1. Symptoms and Diagnoses

**Figure 3:**
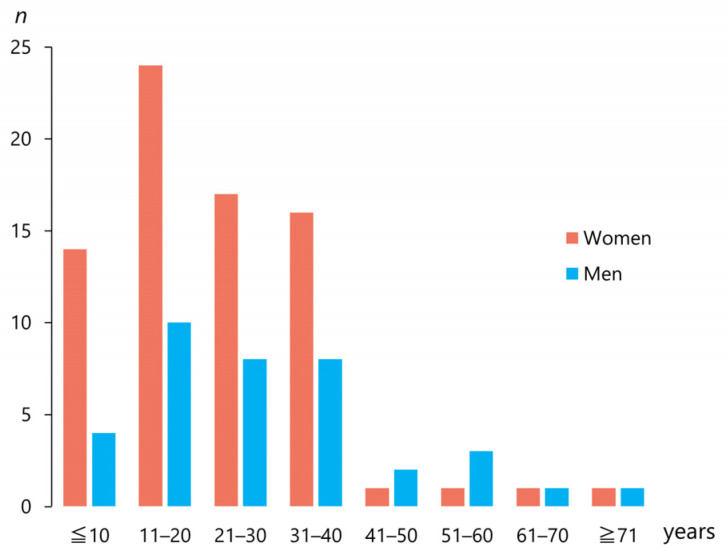
Number of men and women with traumatic superior dislocation discriminated by age group.

The 104 articles included 116 patients (mean age ± standard deviation; 25.7 ± 15.9 years; range, 5–81 years, median, 22 years). The patients were 78 women (67.7%) and 38 men (33.3%; Table 1). Figure 3 shows the number of men and women with a traumatic superior dislocation (*n* = 112) discriminated by age group. Forty patients (34.5%) were aged <18 years. A female majority was observed up to 40 years of age but not beyond (Figure 3). Among the cases, 51 (44.3%) were left-sided, 62 (63.9%) were right-sided, and only one (0.9%) was bilaterally affected [64] (Table 1). 

**Figure 4:**
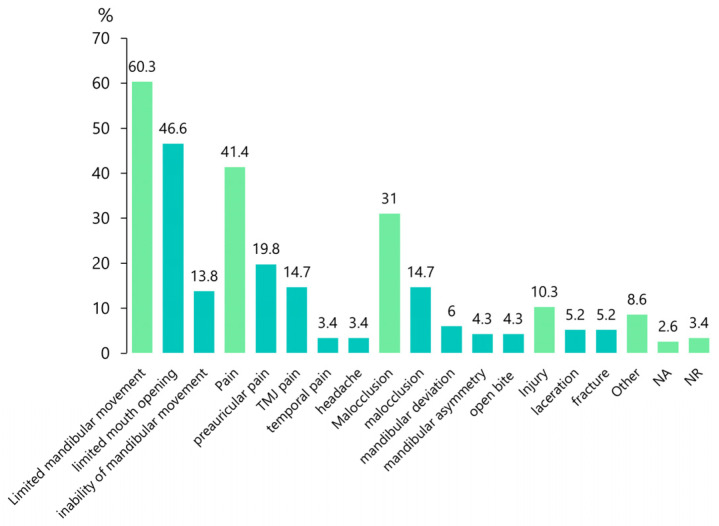
Chief complaints reported by the patients. NA, not applicable; NR, not reported; TMJ, temporomandibular joint.

The most frequent chief complaint was limited mandibular movement (60.3%), followed by pain (41.4%), malocclusion (31%), and injury (10.3%; Figure 4). Demographic data and diagnostic results are summarized in Table 1.

Until the 1990s, radiography was the mainstream diagnostic imaging modality, and tomography began to be used in the 1960s (Table S1). Since the late 1980s, the use of computed tomography (CT) has increased. Magnetic resonance imaging (MRI) has also been used to evaluate neurological problems, such as intracranial hemorrhage. CT was the most frequent diagnostic imaging modality, used in 76 cases (65.6%), followed by a radiography in 69 cases (59.5%) and MRI in 10 cases (8.6%; Table 1). The diagnosis was confirmed with CT in 76 cases (65.6%; Table 1), followed by tomography in 30 cases (25.9%), radiography in 9 cases (7.8%), MRI in 7 cases (6%), and autopsy in 4 cases (3.4%; Table 1).

Total intrusion of the condyle was observed in 56 cases (48.3%), whereas it only intruded but was not incarcerated in 48 cases (41.4%; Table 1).

Forty-six patients (39.7%) were diagnosed immediately (Table S1). The mean delay to diagnosis was 304.8 ± 1986.8 days (median, 2 days; Table 1), and the longest delay was 19,710 days (54 years) [94].

The patients’ symptoms are summarized in Table 2. Mean maximal mouth opening at the first visit was limited to 10.6 mm. The mandible deviated in 77 patients (66.4%), and an open bite was observed in 61 patients (52.6%; Table 2).

Neurological symptoms included the loss of consciousness (19.8%), otorrhagia (14.7%), hearing loss (13.8%), intracranial hematoma (17.2%), dural tear (14.7%), and cerebrospinal fluid leak (3.4%). Other symptoms included a chin laceration (20.7%), cerebral contusion (12.9%), facial nerve paralysis (11.2%), facial injury (9.5%), and pneumoencephalopathy (8.6%; Table 2). 

A summary of fractures other than those of the glenoid fossa is presented in Table 3. Other orofacial fractures included the condyles [21.6%: (ipsilateral, 12.1%; contralateral, 7.8%), symphysis (12.1%), ramus (5.2%), body (5.2%), and angle (3.4%; Table 3).

Fractures other than those in the orofacial region occurred in the ribs (5.2%), limbs (5.2%), skull base (4.3%), femur (3.4%), and clavicle (2.6%; Table 3).

### 3.2. Etiology

**Figure 5:**
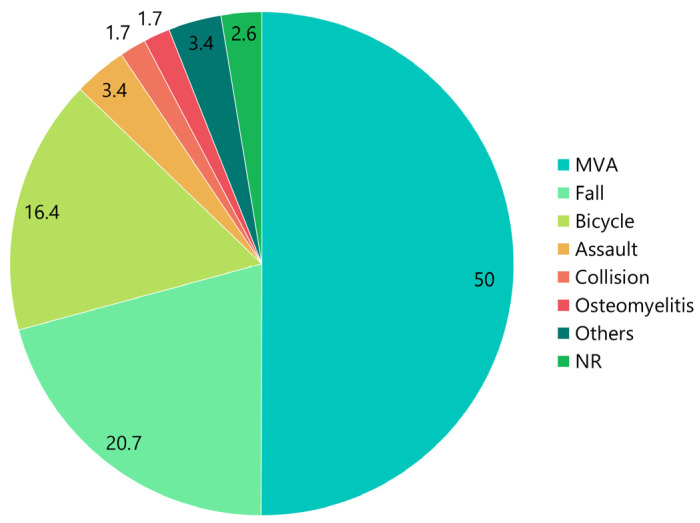
Etiologies of patients with superior dislocation of the condyle into the middle cranial fossa. The numbers in the pie chart represent percentages. MVA, motor vehicle accident; NR, not reported.

The percentages corresponding to each etiology in all patients are shown in Figure 5. The etiologies were motor vehicle accidents (MVA; 50%, *n* = 58), falls (20.7%, *n* = 24), bicycle accidents (16.4%, *n* = 19), assault (3.4%, *n* = 4), collision (1.7%, *n* = 2), osteomyelitis (1.7%, *n* = 2), and others (3.4%, *n* = 4). Other etiologies included industrial accidents [14], accidents [80], degenerative changes of TMJ [107], and orthognathic surgery [108]. The etiology was not reported in three cases (2.6%).

**Figure 6:**
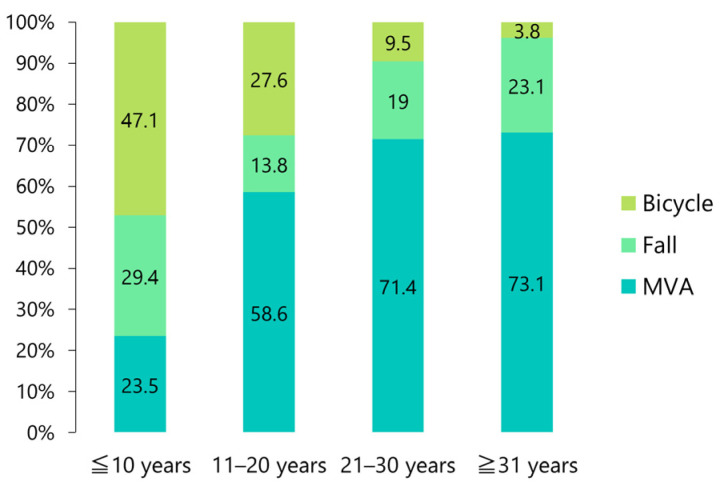
The relative percentage of the three most common etiologies for superior dislocation of the condyle into the middle cranial fossa, discriminated by age.

Approximately half (47.1%) of those under the age of 10 years had bicycle-related accidents; however, this number decreased with increasing age, with only 3.8% among those aged 31 years and older (Figure 6). An MVA was the most frequent etiology in patients aged 31 years and older (73.1%; Figure 6). 

**Figure 7:**
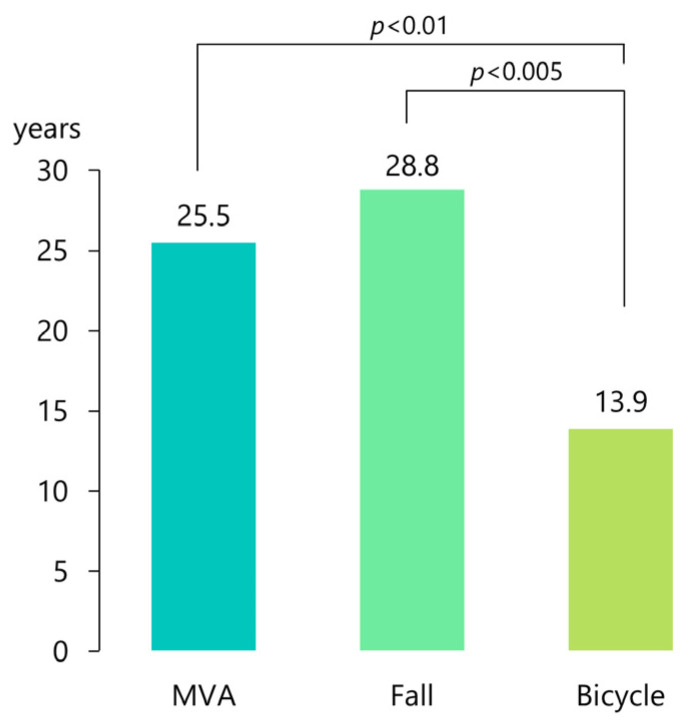
Mean patient age for each of the three most common etiologies of superior dislocation of the condyle into the middle cranial fossa. MVA, motor vehicle accident.

The mean age of patients who suffered a dislocation due to a bicycle accident (*n* = 19) was 13.9 ± 7.5 years (median, 11 years), and they were significantly younger than those who sustained the injury after a fall (28.8 ± 21.9 years; median, 23 years; *n* = 24; *p* < 0.002, analysis of variance) and after an MVA (25.4 ± 11.3 years; median, 23 years; *n* = 58; *p* < 0.01, analysis of variance; Figure 7). 

### 3.3. Treatments and Sequelae

Table S2 shows the results of treatment, complications, follow-up, and sequelae in all patients. Table 4 summarizes the treatment of the cases. Traction using splints or wire was attempted in 18 patients (15.5%); however, it was successful in only 2 cases [6,75] (Table 4). Closed reduction was attempted in 47 patients (40.5%) and was successful in 22 (19%) (Table 4). 

Closed reduction was successful in 29 patients (25%), whereas 66 patients (56.9%) required an open procedure (Table 4). Manual reduction was possible in 18 patients (15.5%). However, 23 patients required TMJ surgery, such as condylotomy, condylectomy, or TMJ prosthesis (Table 4). Of the 66 patients who required open reduction (open manual reduction, 18 [15.5%]), 25 (21.6%) required a craniotomy. Nine of the patients required a TMJ surgery (Table 4). This condition was conservatively observed in four cases [14,94,107,108]. New dentures were inserted in two cases [12,15]. The average delay until treatment was 165.2 days, and it ranged from immediate treatment to 5052 days (13 years and 10 months) [10]. Eleven patients (9.5%) were treated immediately. Regarding the approach used during the open procedure, a preauricular incision (31.9%) was the most frequent, followed by temporal (8%), intraoral (6%), and coronal (4.3%) incisions (Table 4). 

Surgical procedures performed on the TMJ included a condylectomy (12.9%), condylotomy (8.6%), repositioning of the disk (6%), removal of injured tissues (5.3%), and TMJ reconstruction (3.4%). Various combinations of craniotomy and TMJ surgery were performed according to the needs of each patient (Table 4). The glenoid fossa was reconstructed using a bone graft (15.5%), temporalis muscle (5.3%), and temporalis fascia (4.3%).

Treatment complications included facial nerve paralysis (6.9%) and redislocation (2.6%). No complications were observed in 77 patients (66.4%; Table 5). An intermaxillary fixation after reduction was performed in 71 patients (61.2%), with a mean duration of 20.1 days (Table 5). Physical training was performed in 44 cases (37.9%) for an average of 4.5 months (Table 5). After reduction, six patients received orthodontic treatment, and four patients underwent orthognathic surgery. The follow-up was 21.6 months on average, ranging from 2 weeks to 10 years [105], and was not reported in 19 cases (16.4%). At follow-up, the maximal mouth opening increased significantly from 10.6 mm before treatment to 36.2 mm after treatment (*p* < 0.001, paired *t*-test; Table 5). A deviation was observed in 45 patients (38.8%). The sequelae included degenerative changes of the condyle (12.1%), facial asymmetry (5.2%), and death (3.4%) and were not reported in 84 cases (72.4%; Table 5).

**Figure 8:**
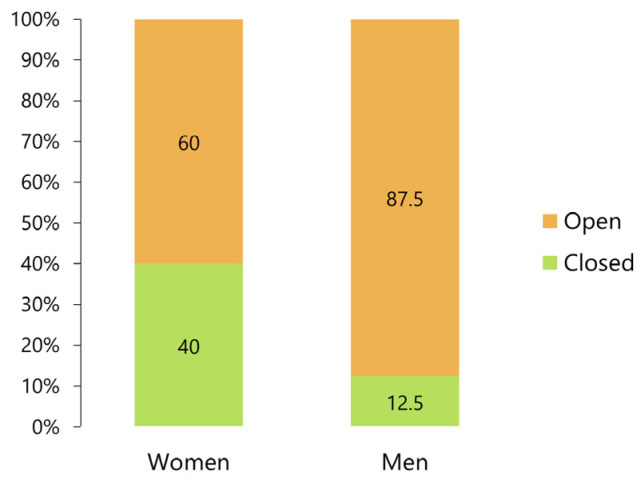
Comparison of the frequency of closed and open reduction procedures in women and men.

Among the patients, 60% of the women required open reduction and 40% required closed reduction, whereas 87.5% of the men required an open procedure (Figure 8). The difference was statistically significant (*p* < 0.01, Fisher’s exact test).

**Figure 9:**
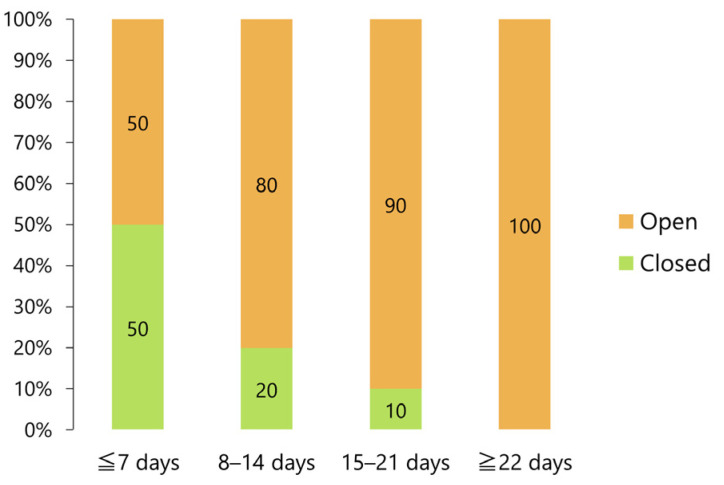
Relative frequencies of closed and open reduction procedures according to time lapsed after injury.

The ratio between closed and open reduction procedures was the same within 7 days after injury; however, the proportion of closed procedures significantly decreased over time, with open reduction required in 90% of the cases at 15–21 days after injury (*p* < 0.05, Fisher’s exact test) and in all cases at 22 or more days after injury (*p* < 0.001, Fisher’s exact test; Figure 9).

**Figure 10:**
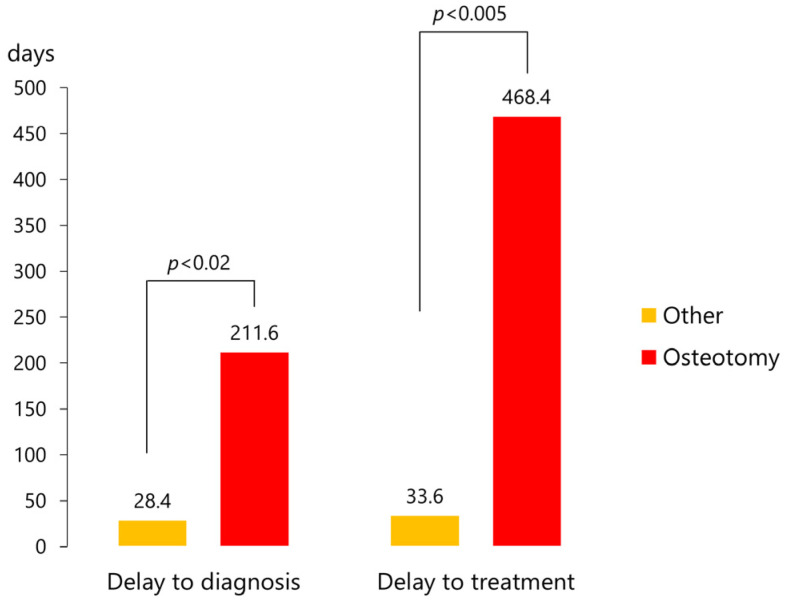
Delays to diagnosis and treatment in cases that did or did not require osteotomy.

Cases requiring osteotomy (*n* = 28) had significantly longer diagnosis delays (211.6 ± 542.4 days [median, 10 days] versus 28.4 ± 141.9 days [median, 0 day]; *p* < 0.02, unpaired *t*-test) and a delay to treatment (468.4 ± 1089.7 days [median, 54 days] versus 33.6 ± 147.3 days [median, 3 days]; *p* < 0.005, unpaired *t*-test) than the other cases (*n* = 72) (Figure 10).

**Figure 11:**
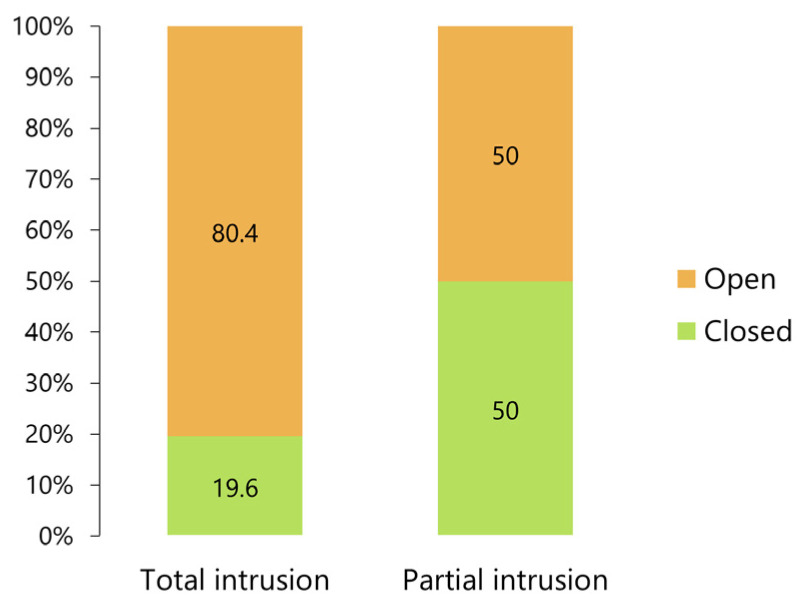
Frequencies of closed and open reduction procedures in patients presenting total or partial intrusion.

Eighty percent of patients with total intrusion of the condyle required open reduction, and the frequency of closed and open reduction procedures in those who presented only an intrusion was comparable (Figure 11). The difference in the proportions was statistically significant (*p* < 0.005, Fisher’s exact test).

**Figure 12:**
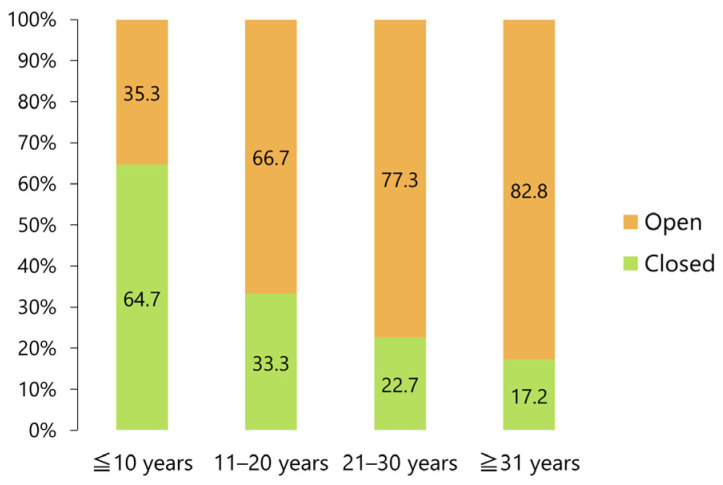
Frequency of closed and open reduction procedures discriminated by patient age.

A closed reduction was possible in approximately two-thirds (64.7%) of patients under 10 years of age, although the percentage significantly decreased with age, reaching 17.9% in patients aged 31 years or above (*p* < 0.005, Fisher’s exact test; Figure 12). 

Results of the binomial logistic regression analysis are summarized in Table 6. The analysis revealed three factors: sex, treatment delay, and degree of penetration. In other words, open reduction was significantly more frequent in men and in patients with longer delays until treatment and less frequent in patients presenting an intruded condyle with partial intrusions (Table 6).

## 4. Discussion

This is the first comprehensive review of all existing reports on superior dislocation of the mandibular condyle into the cranial fossa. Thus, this review included more than two or three times as many patients as previous reviews on the subject. The results of the binomial logistic regression analysis revealed that open reduction was significantly more frequent in men (*p* = 0.026, odds ratio: 4.959) and in patients with a longer time interval from trauma until treatment (*p* = 0.027, odds ratio: 1.124) and less frequent in patients affected by a partial intrusion (*p* = 0.011, odds ratio: 0.186). Therefore, early diagnosis based on careful radiological investigations, such as CT and MRI, is essential to guarantee minimally invasive treatment.

### 4.1. Diagnosis of the Condyle and Glenoid Fossa

A thorough search of electronic medical literature databases combined with a manual search revealed 116 cases of a superior dislocation of the condyle into the cranial fossa. This entity is considered extremely rare owing to anatomical and biomechanical factors. However, many severely injured patients die because of fatal neurological damage, and the actual prevalence is likely to be much higher. This review included three cases in which intracranial displacement was confirmed during an autopsy after death due to intracranial injuries, such as an epidural or subdural hematoma or hemorrhage [28,99]. Additionally, in mild cases, clinical signs may be subtle and likely to be overlooked without a thorough examination.

**Figure 13:**
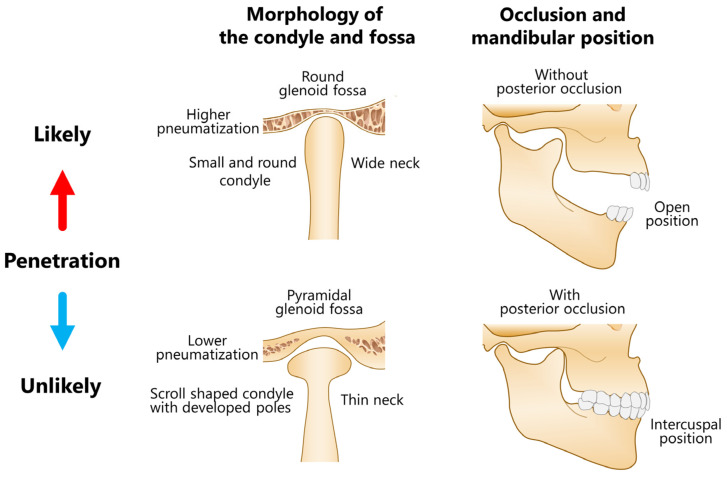
Safety mechanism of the condyle and glenoid fossa and predisposing factors for superior dislocation of the condyle into the cranial fossa.

A central dislocation of the condyle occurs rarely owing to a safety mechanism that protects the cranium and brain from penetration by the condylar head [14]. Typically, the condyle is fractured at the neck. Several predisposing factors for condylar intrusion have been reported. A small round condyle with a wide neck resulting from underdeveloped medial and lateral poles and round fossa are more likely to result in penetration compared to a scroll-shaped condyle with well-developed poles with a thin neck and pyramidal fossa [118] (Figure 13). The former is often observed in pediatric patients, whereas the latter is more frequent in adults. In the adult condyle, the distance between the two poles tends to be greater than that of the glenoid fossa. The thicker portions of a pyramidal glenoid fossa serve as a buttress to dilute the force. A small round condyle with a wide condylar neck can lead to a superiorly directed impact on the roof of the glenoid fossa, which is the weakest and thinnest part of the central bony lamina, when violent force is applied to the chin. Yale et al. [119] evaluated 251 mandibles and classified them into 4 condylar types (flattened, concaved, angled superiorly, and rounded) based on the morphology of the superior surface. The rounded type was observed in 11.8% of cases [119]. Although the number of cases in patients under 20 years of age was low (*n* = 8), 50% of them belonged to the rounded type. Furthermore, patients without posterior molar support [3] during mouth opening were more likely to experience this type of injury [40,118] (Figure 13). However, whether occlusal support at the molars can resist an upward-directed force on impact remained speculative. The mouth would likely have to be forced wide open at maximum impact [29]. Moreover, the disk together with the contiguous muscle structures (including ligamentous attachments) may dissipate the impact force [118]. 

Approximately half of the injuries in patients under the age of 10 years were caused by bicycle accidents (Figure 6), suggesting that an intracranial dislocation may occur even in the absence of a high-energy impact (such as in an MVA). Women exhibited a significantly higher rate of closed reduction than men (Figure 8). This might be because, similarly to pediatric patients, women were more likely to suffer a superior dislocation than men. Dahlberg et al. [43] compared the distribution of sexes in their reviewed data with that observed in a different dataset for other types of facial and mandibular fractures and found that women were significantly overrepresented in their data. In this review, 67.2% of the patients were women, confirming a bias toward the female gender for this particular kind of injury. Without posterior occlusal support, particularly at an open position, a high-velocity blow to the chin is directed superiorly through the roof of the glenoid fossa [40,118]. High pneumatization is also considered a predisposing factor [3,65] (Figure 13); however, confirming this assertion in the present analysis was not possible owing to the lack of objective data. A recent review on pneumatization of the articular eminence [120] showed that the overall prevalence of articular tubercle pneumatization was 25.22% (*n* = 6393, women: 25.14%, men: 25.81%), showing no correlation between the frequency, location, or type of pneumatization and the age or sex of the patient. The average minimum thickness of the glenoid fossa roof was approximately 0.8 mm and was not significantly correlated with sex, age, or mandibular head morphology [121,122]. No obvious reason was available for the prevalence of women among patients affected by this type of injury. Facial fractures are generally considered uncommon in pediatric populations because of the elasticity of the developing poles and relatively wide condylar necks, making the high incidence observed in children difficult to explain. Therefore, a more detailed analysis with a larger number of cases is necessary.

Radiography was the mainstream diagnostic imaging modality until the 1990s, although tomography began to be used in the 1960s. However, this condition was difficult to diagnose in some cases due to unclear imaging, which might have contributed to a misleading initial diagnoses. Since the late 1980s, the use of CT has greatly improved the accuracy of diagnosis compared with conventional radiography. The development of CT and three-dimensional reconstruction allows a clear visualization of the central dislocations, facilitating a definitive diagnosis. A CT scan, particularly in the coronal view, is considered the gold standard for diagnostic imaging of the condition. This study suggested that whether the mandibular condyle penetrates the base of the skull (that is, whether there is a total intrusion) determines the optimal treatment strategy; therefore, detailed visualization using CT is indispensable. CT should be performed immediately after the reduction procedure to confirm the correct position of the condyle within the glenoid fossa and rule out iatrogenic injuries. Koretsch et al. [50] recommended performing CT immediately after surgery, followed by a second scan 24 h later. 

With clear diagnostic imaging and prior knowledge of the superior condylar dislocation, reaching a diagnosis is not particularly difficult; however, some challenges may be present in certain cases. CT scans from a patient affected by the condition had been reported by four services participating in the initial care, but no correct diagnosis was made [49]. In this review, only 39.7% of the patients were immediately diagnosed as having an intracranial displacement. However, this condition was occasionally misdiagnosed as a condylar neck fracture or anterior dislocation of the condyle. As the latter two conditions are overwhelmingly frequent, carefully excluding them during the first examination step is necessary. Common clinical features include limited mandibular movement (60.3%: limited mouth opening [46.6%], mandibular movement inability [13.8%]), pain (41.4%: preauricular pain [19.8%], TMJ pain [14.7%], temporal pain [3.4%], headache [3.4%]), and malocclusion (31%: malocclusion [14.7%], mandibular deviation [6%], mandibular asymmetry [4.3%], open bite [4.3%]; Table 1). These findings were similar to those reported for a unilateral condylar fracture, which could lead to a misdiagnosis during the first examination. Some authors [29,55,64] have reported that approximately half of the patients with a superior dislocation have been misdiagnosed and have had proper treatment delayed as a consequence. A penetration injury is not initially considered because of its relative rarity compared to the more prevalent mandibular condylar fracture, and this lack of awareness likely hinders the ability of some practitioners to make a proper diagnosis [49]. Some of these patients were not diagnosed until after failed surgical treatment attempts [29]. 

The neurological symptoms included a loss of consciousness (19.8%), otorrhagia (14.7%), hearing loss (13.8%), intracranial hematoma (17.2%), dural tear (14.7%), and cerebrospinal fluid leakage (3.4%; Table 2). An epidural hematoma was related to laceration or disruption of the middle meningeal artery, and a subdural hematoma was related to the posterior cerebral artery. Metzner et al. [28] reported the case of a patient who died a few hours after falling on the chin. The cause was confirmed at autopsy to be an epidural hematoma caused by the impaction of the centrally displaced condyle [28]. Potential late complications from damage to the temporal lobe include epilepsy, which occurred in more than 50% of the patients who sustained a compound and depressed skull fracture from a penetrating injury [45]. 

### 4.2. Etiology and Treatment of Intracranial Dislocation of the Condyle

Common etiologies of an intracranial dislocation of the condyle included MVAs (50%), falls (20.7%), bicycle accidents (16.4%), and assault (3.4%; Figure 5). The main cause of this condition are high-energy traffic accidents. The forces associated with an MVA have a much higher impact than those associated with bicycle accidents. Previously, the reported cases were those associated with trauma; however, cases linked to causes other than trauma have recently been reported. These include chronic osteomyelitis [77,109], degenerative changes in the TMJ [107], and asymptomatic intracranial intrusion after orthognathic surgery [108]. A chronic mandibular and/or middle skull base osteomyelitis with a concurrent brain abscess can result in a non-traumatic intracranial condylar head displacement [109]. Skármeta et al. [123] also reported a patient with destructive monoarticular arthritis who developed erosion of the condyle and attrition of the superior articular fossa, resulting in communication within the cranium.

Treatment modalities vary widely, from no treatment with close observation to closed reduction under general anesthesia or a craniotomy with intracranial condylectomy. Four patients included in this study were not provided treatment and were closely observed instead [14,94,107,108]. Elastic splint traction was successful in two cases [6,36]. Injuries due to an MVA tended to be treated with open reduction, whereas those related to bicycle accidents were more frequently treated with a closed procedure. Arya and Chigurupati [87] indicated four factors that influence the choice between open versus closed reduction: (1) age of the patient, (2) time from injury to diagnosis, (3) associated intracranial and neurological injuries, and (4) etiology of the injury. The necessity for open reduction can depend on neurological injuries, including a cerebral contusion or intracranial epidural and subdural hematoma [87]. The aim of the reduction of a dislocated and intruded condyle is to restore the occlusion and mandibular functions, such as chewing and speaking as well as facial appearance; prevent and minimize additional neurological symptoms; and avoid future TMJ ankylosis or facial asymmetry. Closed reduction should be attempted within two weeks of the trauma without an ipsilateral condylar fracture. Blind manipulation can damage the middle meningeal artery and cause intraparenchymal bleeding. Therefore, consulting a neurosurgeon is necessary to determine whether manipulation to reposition the intruded condyle is contraindicated. Fortunately, no cases of intracranial bleeding following reduction have been reported. In addition, no complications were noted in 77 patients included in this study (66.4%; Table 5). Closed reduction could be performed manually or with other instruments (Table 4 and Table S2). Several researchers have used other instruments, such as the Molt mouth prop [18], cap splints and wire [32], Erich arch bars and towel clips [34], Fergusson gag [36], steel bar [41], Schuchardt splint [46], and bite block [53]. The Hippocratic method is the most common technique for manual reduction in an anterior dislocation [112]. The physician places the thumb laterally next to the teeth and the other fingers on the lower surface of the mandible and exerts pressure, first caudally and then dorsally [110,124]. Performing a forceful manipulation for a superior dislocation three weeks or later after the initial trauma might cause additional iatrogenic cerebral damage or enlarge the defect due to adherent bony fragments, resulting in intracranial bleeding due to injury of the middle meningeal artery. Although many authors considered a period of four weeks or less as the criterion for attempting closed reduction, this review found no case in which closed reduction was successful after 22 days from the original trauma (Figure 10). Beyond two to four weeks, enough early healing has occurred, which renders closed reduction ineffective or unstable [49]. Therefore, closed reduction should be attempted in patients only within three weeks from the original injury.

After successful reduction, intermaxillary fixation is required to maintain occlusion and prevent dislocation recurrence. Redislocation has been reported by several researchers [40,64,78,92]. However, maxillomandibular fixation should be as brief as possible to minimize the risk of ankylosis. Ihalainen and Tasanen [27] have suggested that no intermaxillary fixation is necessary in young persons. Intermaxillary fixation after reduction was conducted in 71 patients (61.2%), with a mean duration of 20.1 days (Table 5). Fixation should be followed by physical training or rehabilitation to restore occlusion, jaw function, and mouth opening ability. Physical training was performed in 44 cases (37.9%) for 4.5 months on average (Table 5).

Open reduction should be considered when (1) neurological symptoms associated with a comminution of the temporal bone or cerebral injuries are evident; (2) closed reduction has failed; (3) more than two weeks have elapsed since the original trauma; (4) consolidation, healing, and adhesion have occurred; (5) a coexisting ipsilateral condylar fracture has occurred; (6) there is a high risk of hemorrhage or parenchymal injury with manipulation; and (7) a bony interference is present between the intruded condyle and the comminuted fragments of the glenoid fossa [49,64,66,73,87]. A concomitant fracture of the condylar neck may hinder closed reduction. Open reduction can be performed either extracranially or intracranially, making a close cooperation between neurosurgeons and maxillofacial surgeons essential for an open procedure. A condylectomy, condylotomy, or craniotomy should be considered as alternatives if manual reduction proves unfeasible owing to bone adhesion or the consolidation in the extracranial approach. Various combinations of craniotomy and osteotomy were performed according to the symptoms, risks, and indications of individual patients (Table 4). In cases with a late diagnosis, the dislocated condyle and fragments of the fossa can be adhered, and forceful reduction can result in an intracranial injury. Therefore, a condylectomy [9,10,17,26,68,89], condylotomy [4,7,44,49,52,63,69], or gap arthroplasty [5,33,60,95] is required. 

After open reduction, the glenoid fossa should be reconstructed to ensure separation from the cranial cavity and re-establish a functional articulation using autogenous or alloplastic material, including bone [17,35,38,42,45,49,51,55,56,57,58,59,66,68,80,84,97,98,102], cartilage [52,58,60,69], temporalis fascia [29,37,64,71,80,95,101,109], temporalis muscle [62,63,73,96,103,106], titanium plates [40,47,81,88,89], silicon [22,37], Silastic [16], Gelfoam [7], or Duragen [86] (Table S2). Reconstruction of the condyle is required to restore ramus height when a condylectomy or condylotomy has been performed. The goals of glenoid fossa reconstruction are to prevent recurrent dislocation, re-establish posterior facial height, and restore normal TMJ function [49,66]. The selection of a reconstruction material should be based on the age of the patient, time elapsed between initial injury and treatment, and size of the defect. TMJ prostheses are becoming increasingly popular, and they were applied in four cases included in this review [72,76,90,93]. A recent systematic review concluded that TMJ prostheses are reserved for patients presenting with persistent pain, bony or fibrous ankylosis, or osteomyelitis after the primary closed or open treatment of mandibular condyle fractures [125]. Although the potential consequences of closed reduction in pediatric patients later in life remained unclear, the procedure should be attempted. Open procedures, including condylectomy and condylotomy, should be avoided to prevent facial asymmetry resulting from potential future disturbances to the mandibular growth.

This review revealed that open reduction was significantly more frequent in men and in cases with a longer interval between injury and treatment, and it was less frequent in cases without incarceration. This result indicated that an early definitive diagnosis based on adequate diagnostic techniques is indispensable to guarantee a minimally invasive treatment. However, in several cases [15,38,49,84], the diagnosis or treatment of the dislocation were delayed due to other life-threatening injuries or conditions. Patient’s age, presence of neurological symptoms, and etiology might influence treatment decisions and contributed to treatment outcomes. However, statistical differences was not detected in this study due to the number of patients included (*n* = 116). Further research that includes more cases is required for the proper analysis of significant factors affecting the outcome. 

Despite the absence of neurological deficits, a dural tear was detected in some cases [59,66,81,88], suggesting that consultation with a neurologist or neurosurgeon is necessary for the evaluation and treatment of neurological symptoms. Furthermore, having a neurosurgeon on stand-by during the reduction procedure would be indispensable owing to the possible occurrence of intracranial complications, such as cerebrospinal leakage or intracranial bleeding. Additionally, when patients presented with otorrhagia or hearing loss, a consultation with an otorhinolaryngologist would be essential. Oral surgeons or orthodontists may also play a role to evaluate the change in occlusion, TMJ function, and facial asymmetry during the follow-up period. Therefore, a multidisciplinary team approach that includes specialists in neurosurgery, neurology, otolaryngology, traumatology, anesthesiology, oral and maxillofacial surgery, and orthodontics should be preferred for the evaluation, diagnosis, treatment, and long-term follow-up of these patients.

### 4.3. Limitations and Future Directions

Recently, a consensus and evidence-based recommendations for the management of anterior condylar dislocations were published by the European Society of Temporomandibular Joint Surgeons [111]. According to the recommendations, manual reduction should initially be attempted following the Hippocratic method. If the attempt was unsuccessful, a further attempt should be made under medication (muscle relaxants and/or analgesics), and, if required, under analgo sedation or general anesthesia [111]. In cases of recurrent, longstanding, and/or habitual dislocations, securing methods should be considered [111]. Non-surgical methods should have failed before attempting any minimally invasive or open surgical intervention [111]. The use of botulinum toxin for the treatment of a recurrent dislocation [126,127,128] should be considered as a potential indication. An indication for an open surgical treatment [129,130,131] should be established only after the failure of non-surgical treatments and/or a minimally invasive therapy for an anterior condylar dislocation [111]. 

Two different reports [84,87] have proposed algorithms for the management and treatment of a superior dislocation based on previous reports and personal experiences of the authors. Nevertheless, neither general consensus nor sufficient evidence is currently available due to the rarity of this condition. A superior dislocation can have life-threatening consequences associated with neurological damage; therefore, it represents an emergency situation at the time of first examination that is more critical than in the case of anterior dislocation. Such cases should be consulted with a neurosurgeon. Delay in diagnosis can greatly influence the choice of a closed or open treatment strategy. Prompt diagnosis is therefore indispensable to minimize the risk of cerebral complications, ensure a minimally-invasive procedure, and avoid long-term sequelae. Thus, the development of a management and treatment algorithm for intracranial dislocation is an unmet need in the medical field. All studies evaluated in the present work were case reports or case series, with the majority (90.4%) being single-case reports. Therefore, standard analyses normally included as part of a meta-analysis, such as synthesis of results, could not be performed. Further detailed data and much larger sample sizes are necessary to improve our understanding of this entity, explore further treatment algorithms, and prevent long-term sequelae.

Since the mandibular condyle is the center of the growth of the mandible, possible growth implications for the growth of the condyle, mandible, and associated structures should be carefully considered [32,51,73]. The condyle, lateral pterygoid muscle that attaches to it, and articular disk are greatly damaged after a central dislocation of the condyle into the cranial fossa, even in cases of successful closed reduction. The negative effects of this damage were likely to have a greater impact on children. Facial asymmetry associated with an undergrowth of the mandibular ramus on the affected side, mandibular deviation, and ankylosis have been reported as long-term sequelae (Table 5 and Table S2). TMJ disorders and ankylosis may occur even after the growth is complete, and an intensive longitudinal follow-up for more than 10 years should be warranted. If such sequelae are observed during follow-up, orthodontic or orthognathic surgery and TMJ treatment, including surgical procedures or total TMJ replacement, may be considered. The mean follow-up duration in the studies included here was only 21.6 months. Surprisingly, 16.4% of the studies did not report a follow-up period. Only one report followed up with the patient for 10 years [105]. The lateral pterygoid muscle is the primary muscle involved in the mouth’s opening and lateral movements [132,133] and is thought to be severely damaged in this type of trauma because of its attachment to the condyle. Therefore, it is reasonable to speculate that the cause of mandibular deviation at the time of opening is the deficiency of the lateral pterygoid muscle as well as the decrease in the mandibular translation. Van der Linden [56] postulated that the lack of mobility is due to the loss of attachment and degeneration of the lateral pterygoid muscle. However, no studies on the electromyographic activity of the lateral pterygoid muscle after a superior dislocation treatment have been reported. Further research on the long-term mandibular deviation, including larger samples, is required to confirm this hypothesis.

## 5. Conclusions

Appropriate diagnostic imaging and a multidisciplinary team approach involving oral surgeons, neurosurgeons, and otolaryngologists are necessary for a prompt diagnosis and minimally invasive treatment of the superior dislocation of the condyle, thus avoiding long-term sequelae.

## Figures and Tables

**Table 1:** Demographic data and diagnosis.

Sex, (*n* [%)]	Women, *n* = 78 (67.2%); men, *n* = 38 (32.8%)
Age (years), [mean ± SD, range, median]	25.7 ± 15.9, 5–81, 22
Affected side, (*n* [%])	Left, *n* = 53 (45.7%); right, *n* = 61 (52.6%); bilateral, *n* = 1 (0.9%); NR, *n* = 1 (0.9%)
Chief complaint, (*n* [%])	Limited mandibular movement, *n* = 70 (60.3%) [limited mouth opening, *n* = 54 (46.6%); inability of mandibular movement, *n* = 16 (13.8%)]; pain, *n* = 48 (41.4%) [preauricular pain, *n* = 23 (19.8%); TMJ pain, *n* = 17 (14.7%); temporal pain, *n* = 4 (3.4%); headache, *n* = 4 (3.4%)]; malocclusion, *n* = 36 (31%) [malocclusion, *n* = 17 (14.7%); mandibular deviation, *n* = 7 (6%); mandibular asymmetry, *n* = 5 (4.3%); open bite, 5 (4.3%)]; injury, *n* = 12 (10.3%) [laceration, *n* = 6 (5.2%); fracture, *n* = 6 (5.2%)]; other, *n* = 10 (8.6%); NA, *n* = 3 (2.6%); NR, *n* = 4 (3.4%)
Diagnostic image, (*n* [%])	CT; *n* = 76 (65.6%); radiography, *n* = 69 (59.5%); MRI, *n* = 10 (8.6%); NA, *n* = 3 (2.6%); N, *n* = 1 (0.9%); NR, *n* = 4 (3.4%)
Method of diagnosis, (*n* [%])	CT, *n* = 76 (65.6%); tomogram, *n* = 30 (25.9%); radiography, *n* = 9 (7.8%); MRI, *n* = 7 (6%); autopsy, *n* = 4 (3.4%); N, *n* = 1 (0.9%); NA, *n* = 3 (2.6%); NR, *n* = 4 (3.4%)
Total intrusion, (*n* [%])	Y, *n* = 56 (48.3%); N, *n* = 48 (41.4%); NA, *n* = 10 (8.6%)
Delay to diagnosis (days), (mean ± SD, *n* [%], range, median)	304.8 ± 1986.8, *n* = 112 (96.6%), range 0–19,710, 2; NR, *n* = 14 (12.1%)

SD, standard deviation; Y, yes; N, no; NR, not reported; NA, not applicable; CT, computed tomography; TMJ, temporomandibular joint; MRI, magnetic resonance imaging.

**Table 2:** Summary of patient symptoms.

Maximal mouth opening at the first visit (mm), (mean ± SD, *n* [%], median)	10.6 ± 6, *n* = 57 (49.1%), 10; NA, *n* = 3 (2.6%); NR, *n* = 56 (48.3%)
Deviation, (*n* [%])	Y, *n* = 77 (66.4%); NA, *n* = 3 (2.6%); NR, *n* = 36 (31%)
Open bite, (*n* [%])	Y, *n* = 61 (52.6%) (anterior, *n* = 51 [44%); posterior, *n* = 9 [7.8%); lateral, *n* = 1 [0.9%]); N, *n* = 3 (2.6%); NA, *n* = 3 (2.6%); NR, *n* = 49 (42.2%)
Loss of consciousness, (*n* [%])	Y, *n* = 23 (19.8%); N, *n* = 72 (62.1%); NR, *n* = 21 (18.1%)
Otorrhagia, (*n* [%])	Y, *n* = 17 (14.7%); N, *n* = 75 (64.7%); NR, *n* = 24 (20.7%)
Hearing loss, (*n* [%])	Y, *n* = 16 (13.8%); N, *n* = 83 (71.6%); NR, *n* = 17 (14.7%)
Intracranial hematoma, (*n* [%])	Y, *n* = 20 (17.2%) (subdural, *n* = 7; epidural, *n* = 6; temporal lobe, *n* = 4; subarachinoid, *n* = 2; others, *n* = 4); N, *n* = 63 (54.3%); NR, *n* = 30 (25.9%)
Dura tear, (*n* [%])	Y, *n* = 17 (14.7%); N, *n* = 16 (13.8%); NR, *n* = 83 (71.9%)
Cerebrospinal fluid leak, (*n* [%])	Y, *n* = 4 (3.4%); N, *n* = 80 (69%); NR, *n* = 32 (27.6%)
Other symptoms, (*n* [%])	Chin laceration, *n* = 24 (20.7%); cerebral contusion, *n* = 15 (12.9%); facial nerve paralysis, *n* = 13 (11.2%); face injury, *n* = 11 (9.5%); pneumoencephalopathy, *n* = 10 (8.6%); headache, *n* = 6 (5.2%); TMJ ankylosis, *n* = 6 (5.2%); amnesia, *n* = 5 (4.3%); preauricular tenderness, *n* = 5 (4.3%); vomiting; *n* = 4 (3.4%); teeth fracture, *n* = 4 (3.4%); cerebral concussion, *n* = 3 (2.6%); drowsiness, *n* = (2.6%); nausea, *n* = 3 (2.6%); others, *n* = 26 (22.4%); N, *n* = 16 (13.8%); NR, *n* = 6 (5.2%)

SD, standard deviation; Y, yes; N, no; NR, not reported; NA, not applicable; TMJ, temporomandibular joint.

**Table 3:** Summary of fractures other than those of the glenoid fossa.

Orofacial fracture (*n* [%])	Condyle, *n* = 25 (21.6%) (ipsilateral, *n* = 14 [12.1%]; contralateral, *n* = 9 [7.8%); bilateral, *n* = 2 [1.7%]); symphysis, *n* = 14 (12.1%) (ipsilateral, *n* = 9 [7.8%); contralateral, *n* = 5 [4.3%]; ramus, *n* = 6 (5.2%) (ipsilateral, *n* = 4 [3.4%]; contralateral, *n* = 2 [1.7%]); body, *n* = 6 (5.2%) (ipsilateral, *n* = 4 [3.4%]; contralateral, *n* = 2 [1.7%]); angle *n* = 4 (3.4%) (ipsilateral, *n* = 2 [1.7%]; contralateral, *n* = 2 [1.7%]); ipsilateral zygoma, *n* = 3 (2.6%); orbital floor, *n* = 3 (2.6%); ipsilateral temporal, *n* = 1 (0.9%); maxillary alveolar, *n* = 1 (0.9%) N, *n* = 71 (61.2%); NR, *n* = 4 (3.4%)
Other fracture, (*n* [%])	Ribs, *n* = 6 (5.2%); limbs, *n* = 6 (5.2%); skull base, *n* = 5 (4.3%); femur, *n* = 4 (3.4%); clavicle, *n* = 3 (2.6%); paranasal sinus, *n* = 3 (2.6%); pelvis, *n* = 3 (2.6%); pterygoid process, *n* = 2 (1.7%); radius, *n* = 2 (1.7%); temporoparietal, *n* = 2 (1.7%); others, *n* = 20 (17.2%); N, *n* = 85 (73.3%); NR, *n* = 8 (6.9%)

Y, yes; N, no; NA, not applicable; NR, not reported.

**Table 4:** Summary of treatments for superior dislocation of the condyle into the middle cranial fossa.

Traction, (*n* [%])	Y, *n* = 18 (15.5%); N, *n* = 64 (55.2%); NA, *n* = 12 (10.3%); NR, *n* = 22 (19%)
Attempt to perform closed reduction, (*n* [%])	Y, *n* = 47 (40.5%); N, *n* = 32 (27.6%); NA, *n* = 12 (10.3%); NR, *n* = 25 (21.6%)
Treatment, (*n* [%])	**Closed reduction**, *n* = 29 (25%) [manual reduction, *n* = 22 (19%); with instruments, *n* = 7 (6%)]
	**Open reduction**; *n* = 66 (56.9%) Open manual reduction, *n* = 18 (15.5%) (manual deduction, *n* = 11 [9.5%]; with instruments, *n* = 7 [6%]) Condylotomy, *n* = 8 (6.9%) Condylectomy, *n* = 5 (4.3%) Custom TMJ prosthesis, *n* = 4 (3.4%) Arthroplasty, *n* = 2 (1.7%) Condylectomy + coronoidectomy, *n* = 1 (0.9%) Gap arthroplasty, *n* = 1 (0.9%) Gap arthroplasty + coronoidotomy, *n* = 1 (0.9%) Coronoidectomy, *n* = 1 (0.9%) **Open reduction + craniotomy**, *n* = 25 (21.6%) Craniotomy + condylectomy, *n* = 5 (4.3%) Craniotomy + condylotomy, *n* = 1 (0.9%) Craniotomy + gap arthroplasty, *n* = 1 (0.9%) Craniotomy + condylectomy + coronoidectomy, *n* = 1 (0.9%) Craniotomy + mandibulectomy + condylectomy, *n* = 1 (0.9%)
	Traction, *n* = 2 (1.7%)
	Conservative, *n* = 4 (3.4%) Fabrication of new dentures, *n* = 2 (1.7%) Other, *n* = 4 (3.4%)
	NA, *n* = 6 (5.3%)
Delay to treatment (days), (mean ± SD, *n* [%], range, median)	165.2 ± 635.7, *n* = 89 (76.7%), 0–5052, 7; NA, *n* = 12 (10.3%); NR, *n* = 15 (12.9%)
Approach, (*n* [%])	Preauricular, *n* = 37 (31.9%); temporal, *n* = 8 (6.9%); intraoral, *n* = 7 (6%); coronal; *n* = 5 (4.3%); hemicoronal, *n* = 3 (2.6%); submandibular, *n* = 3 (2.6%); others, *n* = 4 (3.4%); NA, *n* = 41 (35.3%); NR, *n* = 4 (3.4%)
TMJ surgery, (*n* [%])	Condylectomy, *n* = 15 (12.9%); condylotomy, *n* = 10 (8.6%); repositioning of the disk, *n* = 7 (6%); removal of injured tissues, *n* = 6 (5.3%); TMJ reconstruction, *n* = 4 (3.4%); coronoidectomy, *n* = 3 (2.6%); arthroplasty, *n* = 3 (2.6%); gap arthroplasty, *n* = 2 (1.7%); disectomy, *n* = 2 (1.7%); others, *n* = 5 (4.3%); NA, *n* = 52 (44.8%); NR, *n* = 1 (0.7%)
Glenoid fossa reconstruction, (*n* [%])	Bone graft, *n* = 18 (15.5%) (temporal bone, *n* = 12 [10.3%]; rib, *n* = 2 [1.7%]; others, *n* = 4 [3.4%]); temporalis muscle, *n* = 6 (5.3%); temporalis fascia, *n* = 5 (4.3%); muscle + fascia, *n* = 3 (2.6%); cartilage, *n* = 2 (1.7%); others, *n* = 7 (6%); N, *n* = 4 (3.4%); NA, *n* = 51 (44%); NR, *n* = 7 (6%)

Y, yes; N, no; TMJ, temporomandibular joint; NA, not applicable; NR, not reported.

**Table 5:** Post-treatment follow-up and sequelae in patients affected by superior dislocation of the condyle into the middle cranial fossa.

Treatment complication, (*n* [%])	Facial nerve paralysis, *n* = 8 (6.9%); redislocation, *n* = 3 (2.6%); others, *n* = 3 (2.6%); N, *n* = 77 (66.4%); NA, *n* = 7 (6%); NR, *n* = 17 (14.7%)
Fixation (days), (*n* [%], mean ± SD, range, median)	Y, *n* = 71 (61.2%), 20.1 ± 12.1, 3–42, 14.5; NA, *n* = 11 (9.5%); NR, *n* = 28 (24.1%)
Physical training, (*n* [%])	Y, *n* = 44 (37.9%); NA, *n* = 11 (9.5%); NR, *n* = 61 (52.6%)
Length of training (months), (mean ± SD, range, median)	4.5 ± 5.6, 0.5–21, 2.5
Orthodontic or orthognathic treatment, (*n* [%])	Y, *n* = 10 (8.6%) [orthodontic treatment, *n* = 6 (5.3%); orthognathic treatment, *n* = 4 (3.4%)]; N, *n* = 47 (41.2%); NA, *n* = 9 (7.9%); NR, *n* = 49 (42.2%)
Follow-up (months), (mean ± SD, *n* [%], range, median)	21.6 ± 22.7, *n* = 94 (81%), 0.5–120, 12; NA, *n* = 3 (2.6%); NR, *n* = 19 (16.4%)
Maximal mouth opening at follow-up (mm), (mean ± SD, *n* [%], range, median)	36.2 ± 6.8, *n* = 90 (77.6%), 22–51, 35; NA, *n* = 3 (2.6%); NR, *n* = 23 (19.8%)
Deviation at follow-up, (*n* [%])	Y, *n* = 45 (38.8%); N, *n* = 18 (15.5%); NA, *n* = 3 (2.6%); NR, *n* = 50 (43.1%)
Sequelae, (*n* [%])	Degenerative change of the condyle; *n* = 14 (12.1%), facial asymmetry; *n* = 6 (5.2%), death; *n* = 4 (3.4%), diminished hearing; *n* = 2 (1.7%), other; *n* = 2 (1.7%); N, *n* = 3 (2.6%); NR, *n* = 84 (72.4%)

SD; standard deviation, Y; yes, N; no, TMJ; temporomandibular joint, NA; not applicable, NR; not reported.

**Table 6:** Summary of binominal logistic regression analysis results.

Factor	*p*-Value	Odds Ratio	95% Confidence Interval
Sex	0.026	4.959	1.208–20.365
Delay to treatment	0.033	1.124	1.013–1.246
Degree of intrusion	0.011	0.186	0.051–0.684

## Data Availability

Not applicable.

## Supplementary Materials

The following supporting information can be downloaded at https://www.mdpi.com/article/10.3390/jcm12113781/s1: Table S1: Summary of demographic data and symptoms; Table S2: Summary of treatments and follow-up.
