# Supplementary material for: Superior Dislocation of the Mandibular Condyle into the Middle Cranial Fossa: A Comprehensive Review of the Literature
Source: J Clin Med. 2023 May 31;12(11):3781. doi: 10.3390/jcm12113781 (PMC10253349; doi:10.3390/jcm12113781)
Supplement: Supplementary file 1 [file jcm-12-03781-s001.zip › Revision-J Clin Med-Yoshida-2023-Table S1.pdf]

Table S1. Summary of demographic data and symptoms

Author/Year of publication	Age	Sex	Side	Etiology	Chief complaint	Diagnostic imaging	Method of diagnosis	Total intrusion	Delay to diagnosis (days)	Maximal mouth opening (mm)	Deviation	Open bite	Loss of consciousness	Otorrhagia	Hearing loss	Intracranial hematoma	Dural tear	CSK leak	Other symptoms	Orofacial fracture	Other fracture
Lefevre de Rochefort (1834) [1]	22	M	R	fall after drinking alcohol	inability to open the mouth and R TMJ pain	N	autopsy	NA	160 ?	0	posterior and L	N	NR	Y	N	NR	torn	NR	contusion of the chin and headache	R condylar neck	N
Heidsieck (1960) [2]	38	M	L	MVA	NR	radiography	radiography	NA	56	NR	to the L	NR	NR	NR	Y	NR	NR	NR	cerebral contusion, facial nerve paralysis, and facial laceration	L zygoma	skull base and L clavicle
Dingman & Grabb (1963) [3]	28	W	R	MVA	NR	NR	NR	NA	immediate	NR	NR	NR	NR	NR	NR	NR	NR	NR	cerebral concussion	NR	NR
Doane (1963) [4]	13	W	R	MVA	mandibular deviation, laceration of the chin, and inability to open the mouth	radiography	radiography	Y	7 ?	6	to the R	NR	Y	N	N	N	intact	N	retrograde amnesia, cerebral concussion, drowsiness, and vomiting	N	fourth and fifth ribs
Steinhäuser (1964) [5]	26	W	R	bicycle accident	limited mouth opening and R TMJ pain	radiography	tomography	N	7	13	NR	anterior	N	N	N	NR	NR	N	teeth fracture	N	N
Peltier & Matthews (1965) [6]	18	W	R	MVA	forehead laceration and mandibular fracture with lateral displacement	radiography	radiography	Y	immediate	NR	to the R	NR	N	Y	N	N	intact	N	mild facial nerve paralysis	R mental area	N
Stoltmann (1965) [7]	25	M	R	MVA	inability to open the mouth, malocclusion, and deviation	radiography	radiography	NA	immediate	NR	to the R	NR	Y	NR	NR	NR	a small rent	NR	amnesia	N	N
	25	M	L	MVA	malocclusion and inability to move the jaw	radiography	radiography	Y	immediate	NR	NR	NR	NR	NR	Y	NR	intact	NR	paresis on the L facial nerve and amnesia	N	N
Cernéa et al. (1965) [8]	13	M	R	bicycle accident	limited mouth opening	radiography	tomography	NA	730 (2 years)	13	10 mm to the R	NR	N	N	N	NR	NR	N	pain in the mental region	N	N
Dechaume et al. (1965) [9]	33	M	L	assault	pain in the L temporal region	radiography	tomography	NA	4	5	to the L	NR	NR	NR	Y	NR	NR	NR	N	N	N
Whitacre (1965) [10]	15	W	L	MVA	inability to open the mouth and L preauricular pain	radiography	planogram of the TMJ	Y	10	NR	to the L	NR	NR	NR	NR	NR	intact	NR	NR	N	N
Brons (1967) [11]	40	W	L	NR	pain in the L ear region and masticatory disturbance	radiography	tomography	N	56	NR	to the L	Y	Y	N	N	NR	NR	N	vomiting and retrograde amnesia	N	N
Rowe & Killey (1968) [12]	50	M	R	MVA	inability to move the jaw	radiography	radiography	Y	immediate	NR	to the R	Y	Y	NR	N	NR	intact	NR	N	N	R femur
Pirok & Merrill (1970) [13]	19	M	R	industrial accident	mandibular fracture and laceration, serving the facial vessels and injuring the marginal mandibular branch of the seventh nerve	radiography	laminogram of the mandibular condyle	N	63	NR	to the R	NR	N	N	Y	N	NR	N	displacement of the molars	R angle and maxillary alveolar	N
Lund (1971) [14]	56	W	R	MVA	limited mouth opening	radiography	tomography	N	244	40 mm between the edentulous alveolar ridges	to the L	NR	Y	NR	NR	NR	NR	NR	cerebral contusion	L condyle	ribs, pelvis, and limbs
Seymour & Irby (1976) [15]	64	M	L	fall from a moving truck	limited jaw movement	radiography	tomography	Y	3	NR	to the L	NR	Y	N	Y	NR	NR	NR	bilateral paralysis of the seventh cranial nerve and L paresis of the sixth cranial nerve	L condyle	basilar skull
Pons et al. (1976) [16]	26	M	R	MVA	limited mouth opening	radiography	tomography	Y	several weeks	15	to the R	NR	Y	NR	NR	NR	intact	NR	damage to the diaphragm and spleen	L condyle and R symphysis	L femur, R astragalus, and L clavicle
Kallal et al. (1977) [17]	15	W	R	bicycle accident	inability to move jaw	radiography	tomography	Y	immediate	NR	to the R	anterior	N	N	N	N	NR	N	chin laceration	L condyle	N
Zecha (1977) [18]	25	W	R	bicycle accident	R periauricular pain and inability to close the mouth	radiography	tomography	N	immediate	2	to the R	Y	N	Y	N	N	NR	N	chin laceration	L condyle and symphysis	N
Schneller (1979) [19]	14	W	R	MVA	malocclusion	radiography	tomography	N	10	NR	NR	anterior	N	N	N	NR	NR	NR	paresthesia in the R cheek	N	R fibular head
Iannetti & Martucci (1980) [20]	38	M	NR	fall due to epileptic attack	lacerated and contused wound of the symphysis	radiography	radiography	N	immediate	NR	NR	NR	NR	N	N	NR	NR	NR	N	N	N
Pieritz & Schmidseder (1981) [21]	18	M	R	MVA	displaced mandibular fracture	radiography	radiography	Y	10	NR	NR	anterior	Y	NR	Y	NR	NR	NR	paresis of the R facial nerve and cerebral contusion	L canine region and R petrous temporal bone	N
Lachard et al. (1981) [22]	17	W	R	assault	pain during mouth opening and limited mouth opening	radiography	tomography	N	NR	0	to the R	N	N	N	N	NR	NR	N	tinnitus	N	N
	18	M	R	MVA	pain during jaw movements	radiography	tomography	NA	90	15	to the R	NR	Y	NR	N	NR	NR	N	head injury	R condyle and symphysis	NR

	41	M	R	NR	limited mouth opening	NR	NR	NA	1 year	13	NR	NR	NR	NR	NR	NR	NR	NR	face injury	NR	NR
Ihalainen & Tasanen (1983) [23]	11	W	R	bicycle accident	R TMJ pain	orthopantomography	orthopantomography	N	immediate	2	10 mm to the R	20 mm anterior	N	Y	N	N	NR	N	hemorrhage in the R middle ear	N	N
Metzner et al. (1984) [24]	26	W	R	fall after drinking alcohol	NA	NA	autopsy	N	NR	NA	NA	NA	N	N	N	epidural hemorrhage	torn	NR	chin laceration and vomiting	N	N
Pepper & Zide (1985) [25]	32	W	R	MVA	inability to open the mouth, headache, and R ear pain	radiography	tomography and CT	N	1	NR	to the R	NR	N	N	N	N	intact	N	chin laceration and facial soreness	R condyle	N
Copenhaver et al. (1985) [26]	9	W	R	bicycle accident	limited mouth opening and pain in the R temporal area	radiography and CT	tomography and CT	Y	2	NR	to the R	NR	N	N	N	temporal lobe hematoma	NR	N	laceration at the inferior symphysis	N	N
Rappaport et al. (1986) [27]	17	W	R	MVA	mandibular parasymphyseal fracture	radiography	polydirectional tomography	N	immediate	NR	NR	NR	NR	Y	NR	epidural and subdural hematomas	NR	Y	R facial nerve paralysis	L parasymphysis and R zygomatic arch	R frontal and basilar skull and extremity
Musgrove (1986) [28]	7	W	L	bicycle accident	limited mouth opening	radiography	tomography	N	immediate	3	to the L	anterior	N	N	N	N	NR	N	nausea	N	N
Masaki et al. (1988) [29]	38	W	L	MVA	limited mouth opening	radiography	tomography	NR	14	5	NR	NR	N	Y	N	N	NR	N	N	mandibular body	N
Paulette et al. (1989) [30]	11	W	L	bicycle accident	inability to move the mouth	radiography and CT	tomography and CT	Y	5	5	to the L	R posterior	N	N	Y	N	NR	N	N	N	N
Christiansen (1989) [31]	9	M	R	three-wheeler accident	R TMJ pain	radiography	tomography	N	NR	20	to the L	NR	N	N	N	Y	NR	N	chin laceration, headache, and fracture of teeth	N	N
	35	W	R	MVA	severe pain on the R side	radiography	tomography	N	180	30	to the L	NR	NR	NR	Y	NR	NR	NR	dizziness, constant headaches, and depression	N	N
Baldwin (1990) [32]	10	M	R	collision during football	mandibular deviation and restricted jaw movement	radiography	tomography	N	immediate	5	8 mm to the R	posterior	N	N	N	N	NR	N	vomiting	N	N
Kamiya et al. (1990) [33]	21	M	L	MVA	malocclusion	radiography and CT	CT	Y	1	10	3 mm to the L	anterior	NR	N	N	subdural hematoma	torn	NR	chin laceration	L condylar head	N
Galioto et al. (1991) [34]	33	W	R	MVA	inability to move the mouth, R preauricular pain, and malocclusion	radiography and CT	CT	Y	NR	NR	NR	anterior	NR	Y	N	Y	NR	NR	N	R condyle and symphysis	lower extremities
Marker (1992) [35]	8	W	R	bicycle accident	R TMJ pain	radiography	tomography	N	immediate	NR	to the R	Y	N	N	N	N	NR	N	chin laceration	N	N
Engevall & Fischer (1992) [36]	37	W	L	MVA	limited mouth opening	radiography and CT	tomography and CT	Y	14	impaired	NR	anterior	Y	N	N	N	NR	N	sensory loss on the L mandible	R condyle and symphysis	N
Hamamoto et al. (1992) [37]	24	W	L	MVA	inability to move the jaw	radiography	tomography	N	1	NR	to the L	NR	Y	N	N	N	NR	N	L preauricular swelling and tenderness	N	N
Chuong (1994) [38]	28	W	R	MVA	R preauricular pain and difficulty to move the mandible	radiography and CT	CT	N	immediate	7	to the R	anterior	N	Y	Y	N	NR	N	laceration of the cheek and L mandibular symphysis region	N	N
Dahlberg et al. (1995) [39]	11	W	R	bicycle accident	R TMJ pain on jaw movement	radiography and CT	CT	N	1	reduced	NR	Y	N	N	N	epidural hematoma	intact	N	cerebral concussion, headache, nausea, and drowsiness	N	N
Tornes & Lind (1995) [40]	37	W	R	MVA	mandibular asymmetry and reduced mouth opening	radiography and CT	CT	Y	14	10	to the R	NR	NR	NR	Y	NR	NR	NR	cerebral contusion, multiple lacerations, and teeth fracture	N	N
Sandler et al. (1996) [41]	16	M	R	MVA	intracranial injury and cerebrospinal fluid leak from the R ear	CT	CT	Y	immediate	NR	to the R	anterior	Y	Y	NR	NR	torn	Y	pulmonary edema and coagulopathy, contusion of the inferior gyri of the temporal lobe	N	N
Ide et al. (1996) [42]	60	M	L	fall due to loss of consciousness	malocclusion	radiography and CT	CT	N	immediate	15	NR	Y	Y	N	N	NR	NR	N	pneumoencephalopathy and chin laceration	L condyle	N
Long et al (1997) [43]	38	W	L	MVA	headache, reduced visual acuity in the L eye. and difficulty in mandibular movement	radiography and CT	tomography and CT	Y	15	8	to the L	anterior	N	Y	N	N	NR	N	N	N	R temporoparietal bone
Benech et al. (1997) [44]	22	M	L	MVA	lacerations in the neck, L preauricular region, and gums	radiography and CT	CT	N	NR	NR	Y	Y	Y	Y	Y	NR	NR	Y	N	R body and L angle	N
Melugin et al. (1997) [45]	37	W	L	MVA	L preauricular pain and limited mouth opening	radiography and CT	CT	N	77	10	to the L	anterior	NR	NR	Y	NR	NR	NR	L facial nerve paresis	R parasymphysis and L ramus	basilar skull, L temporal bone, orbital floor, and orthopedic fractures
Koretsch et al. (2001) [46]	6	W	L	MVA	inability to move the jaw	radiography and CT	3DCT	N	1	13	to the L	anterior	N	N	N	subarachnoid hemorrhage	NR	N	L auricular pain and temporal lobe contusion	N	N
DeFabianis (2001) [47]	6	W	L	fall while playing	limited mouth opening	radiography and CT	CT	Y	210	21	to the L	anterior	N	N	N	N	NR	N	laceration at the inferior symphysis	N	N
Hayashi et al. (2001) [48]	19	W	R	MVA	limited mouth opening	CT and radiography	CT and 3DCT	Y	immediate	15	to the R	slight	N	N	N	NR	NR	N	facial paralysis and pneumoencephalopathy	R condyle	N
Davis (2002) [49]	23	W	L	fall due to stumbling	sore chin and odynophagia	radiography and CT	CT	Y	3	NR	NR	anterior	N	N	N	NR	NR	N	L TMJ tenderness and contusion of the temporal lobe	R glenoid fossa	N
Barron et al. (2002) [50]	8	W	R	bicycle accident	difficulty to move the jaw and mandibular deviation	radiography and CT	CT	N	immediate	NR	to the R	NR	N	N	N	N	NR	N	laceration at the submental area	N	N

Spanio et al. (2002) [51]	57	M	L	MVA	L TMJ pain	radiography and CT	CT	Y	immediate	15	to the L	NR	N	N	N	N	intact	N	injury in the mandibular symphysis	N	N
	10	W	L	bicycle accident	L periauricular pain	radiography and CT	CT	Y	5	10	NR	Y	N	N	N	N	NR	N	chin laceration	N	N
van der Linden (2003) [52]	17	M	R	MVA	R preauricular pain and limited mouth opening	radiography and CT	tomography and CT	Y	2 ?	3	to the R	NR	N	N	N	N	intact	N	chin laceration	N	orthopedic fractures
Parthiban et al. (2004) [53]	19	M	L	MVA	limited mandibular movement	CT	CT	Y	immediate	NR	NR	posterior	NR	NR	NR	basal extradural hematoma	lacerated	NR	cut injury on the forehead and cortical contusion	N	N
Nadal et al. (2005) [54]	17	W	L	fall due to loss of consciousness	L TMJ pain and limited mouth opening	radiography and CT	CT	Y	immediate	6	to the L	anterior	Y	N	N	N	injured	Y	N	N	N
Cillo et al. (2005) [55]	7	M	R	MVA	R jaw pain, limited mouth opening, and malocclusion	radiography and CT	CT	Y	immediate	NR	to the R	anterior	N	N	N	N	torn	N	luxation of the R maxillary central incisor	N	N
de Oliveira et al. (2005) [56]	12	W	R	accidental fall	mandibular hypomobility	radiography and CT	tomography and CT	Y	1,825 (5 years)	16	NR	NR	N	N	N	N	NR	N	TMJ ankylosis	N	N
Harstall et al. (2005) [57]	9	W	R	fall from a climbing pole	inability to close the mouth	CT	CT	Y	immediate	9	10 mm to the R	anterior	N	N	N	N	NR	N	pneumoencephalopathy	N	N
Soares et al. (2005) [58]	36	W	L	MVA	headache, malocclusion, and limited mouth opening	panorex and CT	CT	NA	7	9	to the L	posterior	N	N	N	N	N	N	NR	NA	NR
Clauser et al. (2006) [59]	32	W	L	MVA	mandibular asymmetry with malocclusion and reduced mouth opening	CT and MRI	CT and MRI	Y	120	18	to the L	anterior	N	N	N	N	NR	N	occlusal impairment	N	multiple bone fractures
Ohura et al. (2006) [60]	23	W	L/R	fall due to loss of consciousness	bilateral preauricular pain, difficulty to move the mandible, anterior open bite, and asymmetric occlusion	radiography, CT, and MRI	3DCT	L; Y R; N	a few days ?	NR	to the L	7 mm anterior	Y	N	N	bilateral epidural hematoma	intact	N	L facial nerve paralysis and intracranial pneumatocele	R condyle	N
Rosa et al. (2006) [61]	5	W	R	MVA	facial asymmetry and preauricular pain	CT and MRI	3DCT and MRI	Y ?	730 (2 years)	10	to the R	5 mm anterior	N	N	N	N	NR	N	facial trauma	N	N
Magge et al. (2007) [62]	12	W	R	collision with another child	headache and inability to occlude the teeth	CT	CT	Y	1	10	to the R	L posterior	N	N	N	N	torn	N	edema in the brain	N	N
Healy et al. (2008) [63]	43	W	L	MVA	open bite, malocclusion, and inability to open the mouth	CT	CT	N	5	NR	to the L	anterior	N	Y	N	N	NR	N	facial abrasions	L condyle	pelvis, ribs, sternal, femoral, and lumbar transverse process
Taglialatela Scafati et al. (2008) [64]	10	W	L	bicycle accident	limited mouth opening and R preauricular pan	radiography, CT, and MRI	CT and MRI	Y	21	4	to the R	anterior	N	N	N	N	NR	N	headache	bilateral condyle and symphysis	N
Menon & Sinha (2008) [65]	30	W	L	MVA	restricted mouth opening and bilateral preauricular pain	radiography and CT	CT	Y	28	NR	to the L	posterior	Y	Y	N	N	NR	N	lateral dislocation of the R condyle	L subcondyle	fifth, sixth, and seventh ribs and humerus
Lloyd & Sivarajasingam (2010) [66]	20	M	L	MVA	multiple maxillofacial injuries	CT	CT	N	immediate	NR	to the L	NR	N	N	N	N	intact	N	facial lacerations and multiple teeth fractures	R angle	N
Man et al. (2011) [67]	32	W	L	MVA	malocclusion and limited mouth opening	CT	CT	Y	10	NR	to the L	anterior	N	Y	N	N	NR	N	cerebral contusion and facial ruptures	N	N
Hayashi et al. (2011) [68]	18	W	L	MVA	abrasion in the region of the chin, malocclusion, and limited mouth opening	NR	NR	NR	NR	NR	NR	NR	NR	NR	NR	NR	NR	NR	NR	NR	NR
Struewer et al. (2012) [69]	36	M	R	assault	facial and jugular swelling and tenderness on the R TMJ	CT	3DCT	N	immediate	severely reduced	NR	slight	N	N	N	epidural hematoma	NR	N	cerebral concussion, drowsiness, and nausea	mandibular body, ramus, and zygomatic arch	paranasal sinuses, lateral orbital wall and floor, and pterygoid process
Yoshida & Hyo (2012) [70]	5	W	L	fall from a bar	L preauricular pain and malocclusion	radiography, CT, and MRI	CT	Y	immediate	10	8 mm to the L	anterior	N	N	N	N	NR	N	N	N	N
Jiao et al. (2013) [71]	36	M	L	bicycle accident	L preauricular pain, limitation of jaw mobility, open bite, and deviation	CT	3DCT	N	immediate	15	to the L	anterior open bite of 15 mm	Y	N	N	N	NR	N	contusion of the L temporal lobe	N	N
Garcia-Guevara et al. (2013) [72]	33	W	R	MVA	limited mouth opening, pain, and mandibular deviation	CT	CT	Y	immediate	NR	to the R	NR	N	N	N	N	NR	N	N	N	N
Lee et al. (2013) [73]	53	W	L	chronic osteomyelitis	trismus	CT	CT	N	immediate	NR	NR	NR	N	N	N	N	epidural abscess	N	L facial nerve paresis, memory problems, continuous headaches, confusion, dysphagia, and pneumocephalus	L ramus	N
Tutela et al. (2013) [74]	72	W	R	fall from a bed	bilateral TMJ pain	CT	CT	N	immediate	NR	NR	anterior open bite of 15 mm	N	N	N	intraparenchymal hemorrhage	NR	N	chin laceration	L condyle	N

Zhang et al. (2014) [75]	23	W	R	MVA	restriction of jaw movement and R preauricular pain	CT	CT	N	NR	NR	to the R	L posterior	N	N	N	N	NR	N	N	N	N
	39	M	L	accident	difficulty to open the mouth	CT	CT	N	NR	12	to the L	NR	N	N	N	N	NR	N	R dislocation, L preauricular pain, edge-to-edge occlusion, and cross-bite	N	N
Vaezi et al. (2014) [76]	32	W	R	MVA	facial laceration and limited mouth opening	radiography and CT	CT	Y	immediate	NR	to the R	posterior	N	N	N	N	small tear	N	R TMJ tenderness	N	N
Oberman et al. (2014) [77]	10	W	R	bicycle accident	bilateral TMJ pain and malocclusion	CT	CT	Y	immediate	16	to the R	NR	N	N	N	N	NR	N	questionable muffled hearing	N	N
Asai et al. (2014) [78]	17	W	R	bicycle accident	malocclusion and limited mouth opening	radiography, CT, and MRI	CT	N	1	15	to the R	anterior	N	N	N	subdural hematoma	NR	N	chin laceration	N	N
He et al. (2015) [79]	13	W	R	fall during exercise	limited mouth opening and malocclusion	CT	CT	Y	immediate	15	to the R	anterior	N	N	N	N	NR	N	N	N	N
	25	W	R	MVA	limited mouth opening and malocclusion	CT	CT	Y	14	NR	to the R	anterior open bite of 10 mm	N	N	N	N	partial tear	N	scalp contusion	N	N
	22	W	L	MVA	malocclusion	CT	CT	Y	150	NR	NR	NR	N	N	N	N	intact	N	serious brain injury and coma for 2 months	R mandibular body	extensive skull base fracture
Temiz et al. (2015) [80]	35	W	L	fall	limited mouth opening, anterior open bite, and preauricular pain	CT	CT	N	immediate	NR	NR	NR	N	N	N	N	NR	N	chin laceration	N	N
	18	W	R	MVA	difficulty to open the mouth	CT	CT	N	21	NR	to the R	anterior	N	N	N	N	NR	N	R preauricular tenderness	N	N
Zhang et al. (2016) [81]	20	W	R	MVA	jaw pain exacerbated by talking and movement	CT	CT	Y	immediate	NR	NR	NR	Y	NR	NR	N	NR	NR	amnesia and trismus	R condyle and ramus and L ramus and symphysis	maxillary sinus, L occipital condyle, L clivus, inferior mastoid portion of the temporal bone, C1 transverse process, rib, clavicula, sacroiliac, and fibular
Arya & Chigurupati (2016) [82]	15	W	L	bicycle accident	NR	radiography and CT	CT	N	NR	NR	10 mm to the L	anterior open bite of 8 mm	NR	NR	NR	L temporal lobe hemorrhage	NR	NR	L temporal lobe contusion	N	NR
	5	W	L	fall from a bed	facial asymmetry	radiography	radiography	N	NR	NR	to the L	posterior	NR	NR	NR	NR	NR	NR	NR	N	NR
Kanno et al. (2016) [83]	51	M	R	fall after drinking alcohol	reduced ability to open or close the mouth	CT	CT	Y	immediate	NR	to the R	NR	N	N	N	NR	partial tear	N	chin laceration, R preauricular swelling, and cerebral contusion	R condylar head	N
Zamorano et al. (2016) [84]	9	M	R	accidental fall	severe limitation to open the mouth	CT	CT	Y	1,095 (3 years)	6	to the R	NR	N	N	N	N	intact	N	facial asymmetry, lip incompetence, and micrognathia	N	N
Rikhotso & Bobat (2016) [85]	17	M	R	MVA	inability to open the mouth	radiography and CT	CT	N	180	3	NR	anterior	Y	N	N	N	NR	N	bilateral TMJ ankylosis	L condylar head	N
Pinares & Urzúa (2016) [86]	13	W	L	MVA	limited mouth opening	radiography, cone-beam CT, and MRI	cone-beam CT and MRI	Y	2,190 (6 years)	NR	NR	NR	N	N	N	N	NR	N	L TMJ ankylosis	N	N
Yadegari et al. (2016) [87]	10	W	R	MVA	R pain and deviation	radiography and CT	CT	Y	immediate	NR	to the R	anterior	N	N	N	N	NR	N	chin laceration	N	N
Lindell & Thor (2017) [88]	15	W	L	fall due to epilepsy	lateral open bite	CT	CT	N	immediate	NR	NR	lateral	N	N	N	N	NR	N	N	symphysis and bilateral condyle	N
De Mol et al. (2017) [89]	63	W	R	NR	severe limitation of mandibular mobility	CT and MRI	CT and MRI	Y	19,710 (54 years)	16	2 mm to the R	NR	N	N	N	N	NR	N	N	N	N
Liu et al. (2017) [90]	29	W	L	MVA	limited mouth opening, malocclusion, and chin deviation	3DCT	3DCT	Y	35	NR	to the L	anterior	N	N	N	N	NR	N	coma	L zygomatic arch	N
Kurimori et al. (2018) [91]	23	W	L	MVA	anterior open bite and limitation of jaw movement	3DCT	3DCT	Y	immediate	NR	NR	anterior	N	N	N	N	laceration	N	hypovolemic shock	L condylar neck and R parasymphysis	N
	34	M	L	MVA	difficulty to open the mouth	CT	CT	N	2,190 (6 years)	NR	NR	NR	N	N	N	N	NR	N	L TMJ ankylosis	N	N
Monteiro et al. (2019) [92]	22	M	R	MVA	R preauricular pain	CT	CT	Y	immediate	NR	NR	NR	Y	NR	Y	NR	lesion	NR	facial nerve deficit and cerebral contusion	N	L radius
Liau et al. (2019) [93]	22	W	R	fall while skateboarding	R temporal region pain and limited mouth opening	CT	CT	N	immediate	15	NR	anterior	N	Y	Y	N	intact	N	hemotympanum and pneumocranium	N	N
Holz et al. (2019) [94]	36	W	L	fall out of a window	NA	NA	autopsy	Y	NR	NA	NA	NA	Y	NR	NR	subdural hematoma	NR	NR	chin laceration and internal bleeding due to rupture of the thoracic aorta	mandibular body	ribs, pelvis, both arms, and lumbar spine
	81	M	L	fall downstairs	NA	NA	autopsy	Y	NR	NA	NA	NA	NR	NR	NR	epidural, subdural, and subarachnoid hemorrhage	NR	NR	NR	N	N

Chen et al. (2019) [95]	20	M	R	MVA	malocclusion and limited mouth opening	radiography and CT	CT	N	NR	10	9 mm to the R	anterior and L posterior	N	N	N	NR	NR	N	arachnoid cyst	N	R femur, malleolus, L acetabulum, radius, and tibia
Esquenazi et al. (2019) [96]	23	W	L	fall on the curbside	L decreased hearing, nausea, emesis, facial and L jaw pain	CT	CT	Y	immediate	15	NR	NR	N	N	Y	a small area of hemorrhage, subdural hemorrhage	Torn	N	chin laceration, pneumocephalus, and contusion in the L temporal lobe	L condyle	L mastoid process
Kyrpa et al. (2019) [97]	31	W	R	assault	headache, dizziness, limited mouth opening, R ear pain, nausea, and vomiting	radiography and CT	CT	N	1	NR	N	Y	N	Y	N	R temporal lobe intracerebral hemorrhage and subdural hematoma	NR	NR	pneumocephalus	N	N
Romano et al. (2019) [98]	17	W	L	MVA	otorrhagia, epistaxis, and rhinorrhea	CT	CT	Y	immediate	NR	NR	Y	NR	Y	NR	intracranial hemorrhage	Lacera tion	NR	pneumocephalus	L horizontal mandibular branch and R angle	L temporal bone, sphenoid, orbit walls, nasal bones, R pterygoid processes, and walls of maxillary sinuses
Congiusta & Champion (2020) [99]	25	W	R	fall downstairs	NR	NR	NR	NA	NR	NR	NR	NR	NR	NR	NR	temporal lobe intraparenchymal hemorrhage	NR	NR	NR	R parasymphysis and L condyle	NR
Ramani et al. (2021) [100]	20	W	L	MVA	restricted jaw movements and pain in the jaws	CT	CT	N	immediate	anterior	Y	N	N	N	N	N	NR	N	chin laceration and pneumocephalus	N	N
Díez-Suárez & Paredes-Farrera (2021) [101]	13	M	L	bicycle accident	mandibular lock	CT	CT	Y	immediate	anterior open bite of 10 mm	N	N	N	N	N	N	NR	N	multiple facial abrasions and chin laceration	N	N
Zumbrunn Wojczyńska et al. (2021) [102]	77	W	L	degenerative changes of the TMJ	limited mouth opening and L preauricular pain during mastication	CT and MRI	CT and MRI	N	immediate	NR	N	N	N	N	N	N	NR	N	N	N	N
Motazedian et al. (2021) [103]	23	M	L	orthognathic surgery	facial asymmetry	CT	CT	N	immediate	NR	N	N	N	N	N	N	NR	N	N	N	N
D'Hondt et al. (2022) [104]	58	M	L	chronic osteomyelitis	reduced mouth opening	CT and MRI	CT and MRI	N	immediate	N	N	N	Y	N	Y	N	NR	N	L facial nerve paresis	N	N

M, man; W, woman; L, left; R, right; Y, yes; N, no; NR, not reported; NA, not applicable; MVA, motor vehicle accidents; TMJ, temporomandibular joint; CSK, cerebrospinal fluid; CT, computed tomography; 3D, three-dimensional; MRI, magnetic resonance imaging.
