# Supplementary material for: Superior Dislocation of the Mandibular Condyle into the Middle Cranial Fossa: A Comprehensive Review of the Literature
Source: J Clin Med. 2023 May 31;12(11):3781. doi: 10.3390/jcm12113781 (PMC10253349; doi:10.3390/jcm12113781)
Supplement: Supplementary file 1 [file jcm-12-03781-s001.zip › Revision-J Clin Med-Yoshida-2023-Table S2.pdf]

Table S2. Summary of treatments and follow-up

Author/Year of publication	Traction	Attempt to closed reduction	Treatment	Delay to treatment (days)	Approach	TMJ surgery	Glenoid fossa reconstruction	Complication of surgical procedure	Fixation (days)	Physical training	Length of training (months)	Orthognathic or orthodontic treatment	Follow-up (months)	Maximal mouth opening at follow-up (mm)	Deviation at follow-up	Sequelae
Lefevre de Rochefort (1834) [1]	NA	NA	inflammation relief ?	NA	NA	NA	NA	NA	NA	NA	NA	NA	5	NR	NR	died of brain abscess 157 days after the injury
Heidsieck (1960) [2]	NA	NA	NA	NA	NA	NA	NA	NA	NA	NA	NA	NA	NR	NR	NR	NR
Dingman & Grabb (1963) [3]	N	N	open reduction with condylotomy	31	NR	condylotomy	fascia lata graft	N	Y	NR	NR	R step osteotomy and L vertical osteotomy	90	NR	N	NR
Doane (1963) [4]	Y	Y	open reduction with craniotomy and gap arthroplasty	18	preauricular and coronal	gap arthroplasty	NA	N	Y	NR	NR	NA	36	NR	NR	NR
Steinhäuser (1964) [5]	Y	NR	closed reduction with traction by cap-splint	21	closed	NA	NA	NA	14	Y	2	NA	6	37	N	NR
Peltier & Matthews (1965) [6]	Y	Y	first, reduction of mental area; second, open reduction with condylotomy	54 ?	submandibular incision	condylotomy	Gelfoam	N	42	Y	NR	NA	6	NR	minimally to the R	NR
Stoltmann (1965) [7]	Y	N	open reduction with craniotomy and condylectomy	immediate	subtemporal	condylectomy	NA	N	42	NR	NR	NA	60	able to open widely	NR	NR
	Y	N	open reduction with craniotomy and condylectomy	immediate	subtemporal	condylectomy	NA	facial nerve paralysis	42	NR	NR	NA	NR	no symptom	NR	NR
Cernéa et al. (1965) [8]	N	N	open reduction with craniotomy and condylectomy	970	submandibular	condylectomy	NR	N	NR	NR	NR	NR	2.5	40	to R	obvious atrophy of the R mandible
Dechaume et al. (1965) [9]	N	NR	open reduction with craniotomy and condylectomy	5,052 (13 years and 10 months)	preauricular	condylectomy	NR	N	21	NR	NR	NR	20	35	to L	NR
Whitacre (1965) [10]	N	N	open reduction with craniotomy and condylectomy	10	preauricular	condylectomy	NR	N	NR	NR	NR	NR	26	NR	moderately to the L	NR
Brons (1967) [11]	N	Y	fabrication of new dentures	20	intraoral	NA	NA	N	NA	NA	NR	N	18	32	N	occurrence of "nearrose" at the L TMJ
Rowe & Killey (1968) [12]	N	Y	open reduction with condylectomy	NR	extensive preauricular	condylectomy	meniscus	N	14	NR	NR	N	NR	almost normal range	NR	NR
Pirok & Merrill (1970) [13]	Y	N	conservative	NA	NA	NA	NA	N	42	Y	NR	N	18	satisfactory	NR	NR
Lund (1971) [14]	N	N	fabrication of new dentures	60 ?	NA	NA	NA	N	NA	NA	NA	N	24	37 mm between the edentulous alveolar ridges	10 mm to the L	NR
Seymour & Irby (1976) [15]	NR	N	open reduction with condylotomy	10	extensive preauricular	condylotomy	a prosthetic glenoid fossa that was fashioned out of Silastic and wired into position through the zygoma	N	Y	NR	NR	N	2	26	N	NR
Pons et al. (1976) [16]	N	Y	open reduction with craniotomy and condylectomy	255	preauricular and coronal	condylectomy	temporal bone graft	N	14	NR	NR	N	NR	30	slightly to the R	NR
Kallal et al. (1977) [17]	N	Y	closed manual reduction using a Molt mouth prop	5	NA	NA	NA	N	14	NR	NR	genioplasty	42	NR	NR	a foreshortening of the R condyle with flattening of the articular surface
Zecha (1977) [18]	N	Y	closed manual reduction	2	NA	NA	NA	N	7	Y	NR	N	18	34	to the R	NR
Schneller (1979) [19]	Y	Y	closed manual reduction	10	closed	NA	NA	N	3	Y	NR	N	2	24	NR	NR
Iannetti & Martucci (1980) [20]	NR	NR	open reduction using a lateral mouth prop and meniscectomy	5	retrotragal preauricular	meniscectomy	reconstruction using a silicone rubber	N	15	Y	NR	N	NR	NR	NR	NR
Pieritz & Schmidseder (1981) [21]	Y	Y	closed reduction using a retractor to sigmoid notch	10	approach to sigmoid notch	NA	NA	N	NR	NR	NR	NR	19	moderate restriction	NR	R ear deafness
Lachard et al. (1981) [22]	NR	Y	closed manual reduction ?	NR	closed ?	NA ?	NA ?	R facial nerve paralysis	14	NR	NR	NR	12	30	NR	NR
	NR	NR	open reduction with condylectomy	100 ?	preauricular	condylectomy	NR	NR	NR	NR	NR	NR	27	39	to the R	NR
	NR	NR	open reduction with condylectomy?	NR	preauricular ?	condylectomy ?	NR	R facial nerve paralysis	NR	NR	NR	NR	NR	27	NR	NR

Ihalainen & Tasanen (1983) [23]	N	Y	closed manual reduction	immediate	NA	NA	NA	N	N	NR	NR	N	16	35	4 mm to the R	increased degenerative changes in the R condyle
Metzner et al. (1984) [24]	NA	NA	NA	NA	NA	NA	NA	N	NA	NA	NA	N	NA	NA	NA	died 6 hours after the injury
Pepper & Zide (1985) [25]	N	Y	open manual reduction and removal of fractured segment of the condyle	4	preauricular	reconstruction using a Proplast teflon implant	free graft of the temporal fascia and muscle	neuropraxia of the facial nerve	21	Y	6	N	18	excellent function	N	NR
Copenhaver et al. (1985) [26]	N	Y	closed manual reduction	2	NA	NA	NA	N	21	Y	0.5	N	8	35	minimally to the R	slight resorption of the R condyle
Rappaport et al. (1986) [27]	N	N	open manual reduction, reduction of parasymphyseal and zygomatic arch fractures	immediate	intraoral	NA	NA	R facial nerve paralysis	25	NR	NR	N	15	40	minimally to the R	NR
Musgrove (1986) [28]	Y	Y	closed reduction using cap splints and wires (circum-mandibular, transalveolar, and ascending ramus)	3	submandibular	NA	NA	N	15	Y	0.5	N	6	slight limitation	NR	NR
Masaki et al. (1988) [29]	NR	Y	open reduction with arthroplasty	29	preauricular ?	arthroplasty	Proplast fixation	N	NR	Y	NR	N	10	50	NR	NR
Paulette et al. (1989) [30]	N	Y	closed reduction using Erich arch bars and towel clips	7	NA	NA	NA	N	Y	NR	NR	NR	10	full range	slightly to the L	NR
Christiansen (1989) [31]	Y	N	open reduction with craniotomy	NR	coronal and bilateral preauricular	minimal condylar shaving	rib graft	N	42	NR	NR	orthodontic treatment	12	44	very apparent to the L	surgery to correct the deviation in consideration
	N	N	open manual reduction	180	preauricular	NR	NR	N	Y	NR	NR	NR	NR	50	NR	NR
Baldwin (1990) [32]	N	Y	closed reduction using a Fergusson gag	1	closed	NA	NA	N	14	Y	2	NR	24	24	N	degenerative change of the R condyle
Kamiya et al. (1990) [33]	N	Y	open reduction with craniotomy and reduction using a steel wire	2	coronal and preauricular	removal of condyle and disk fragments	silicon plate and temporal fascia	N	30	Y	NR	N	7	45	5 mm to the L	NR
Galioto et al. (1991) [34]	N	N	open reduction with craniotomy and reduction of symphyseal fracture	NR	temporal and preauricular	osteosynthesis of the fractured condyle	temporal bone graft	neuropraxia of the facial nerve	14	Y	NR	N	10	normal	N	NR
Marker (1992) [35]	N	Y	closed manual reduction	immediate	closed	NA	NA	N	14	Y	NR	N	24	29	NR	absorption of osteophyte of the R condyle
Engevall & Fischer (1992) [36]	Y	N	first, craniotomy with extraction of a fractured piece roof of the glenoid fossa and replacement with fascia; second, condylotomy	first, 14; second, 56	temporal and preauricular	transverse osteotomy of the condylar neck	titan plates placed on the zygomatic arch	TMJ dysfunction and anterior open bite	42	NR	NR	maxillary impaction and mandibular advancement	36	36	N	slight tenderness in the TMJ
Hamamoto et al. (1992) [37]	N	Y	closed reduction using a steel-bar placed between the upper and lower molars	1	closed	NA	NA	N	7	Y	NR	N	4	40	to the L	NR
Chuong (1994) [38]	N	Y	open reduction using a periosteal elevator simultaneously using a bone clamp	NR	modified endaural	disk repositioning	outer table cranial graft	N	6	Y	NR	N	2	36	slightly to the R	diminished hearing on the R
Dahlberg et al. (1995) [39]	NR	NR	craniotomy, evacuation of hematoma, removal of a fragment of the glenoid fossa	2	temporal	NA	N	N	NR	NR	NR	NR	24	48	to the R	a discrete facial asymmetry
Tornes & Lind (1995) [40]	Y	N	open reduction with condylotomy and coronoidectomy	720	extraoral	condylotomy and coronoidectomy	N	N	Y	Y	21	N	60	35	to the R	NR
Sandler et al. (1996) [41]	N	N	open reduction with temporal craniotomy, inferior temporal lobectomy, and tracheostomy	1	hemicoronal	NA	repair using temporal bone and ultramicroplate	facial nerve paralysis due to a total common carotid occlusion	NR	Y	NR	NR	0.5	35	NR	NR
Ide et al. (1996) [42]	Y	Y	closed reduction using elastic traction using a Schuchardt splint	1	closed	NA	NA	N	28	NR	NR	N	72	51	to the L	NR

Long et al (1997) [43]	N	NR	open reduction by upward pressure applied to the chin using a piece of rubber	15	preauricular	smoothing of the condylar head and repair of the disk with sutures	T-shaped titanium miniplate adapted across the glenoid fossa	N	NR	NR	NR	N	NR	good condylar movement	N	NR
Benech et al. (1997) [44]	N	N	osteosynthesis of the R body and the L angle fractures	6	NR	NA	NA	N	N	NR	NR	NR	12	no limitation	NR	sporadic tinnitus
Melugin et al. (1997) [45]	NR	NR	open reduction with condylotomy	84	modified endaural	condylotomy	bone graft with freeze-dried, demineralized, allogeneic rib cortex, and fresh-frozen allogeneic femoral head cartilage	N	NR	NR	NR	N	NR	41	to the L	NR
Koretsch et al. (2001) [46]	N	Y	closed manual reduction	2	NA	NA	NA	N	14	Y	2	N	24	48	to the L	significant resorption of the L condyle
DeFabianis (2001) [47]	N	Y	open manual reduction	210	temporal and preauricular	substitute of the disk by a Gore-Tex membrane	bone graft	N	NR	Y	3	NR	3	29	5.5 mm to the L	a moderate decrease in vertical height of the L mandible
Hayashi et al. (2001) [48]	N	N	open reduction with condylotomy	60	preauricular	condylotomy	auricular cartilage	N	NR	Y	NR	orthodontic treatment	24	40	NR	NR
Davis (2002) [49]	N	Y	closed reduction using a bite block	3	NA	NA	NA	NA	42	Y	NR	NR	36	33	NR	bilateral fibrous ankylosis
Barron et al. (2002) [50]	N	Y	closed manual reduction	immediate	NA	NA	NA	NA	7	NR	NR	NR	24	35	to the R	NR
Spanio et al. (2002) [51]	N	N	open reduction with craniotomy and using a steel wire	20	coronal	temporalis muscle flap between the condyle and the roof of the articular cavity.	temporal bone graft	N	NR	NR	NR	NR	24	37	to the L	NR
	NR	NR	open manual reduction ?	14	preauricular	NA	bone graft using the iliac crest stabilized by a titanium plate fixed to the zygomatic arch	N	NR	NR	NR	NR	8	NR	to the L	NR
van der Linden (2003) [52]	NR	NR	open manual reduction	21	preauricular and temporal	discectomy	temporal fascia graft	N	9	Y	NR	NR	58	36	NR	atrophy and degeneration of the articular surfaces of the R condyle with a fibro-osseous ankylosis
Parthiban et al. (2004) [53]	NR	Y	open reduction with craniotomy by pushing the condyle from in the cranial fossa and pulling the mandible from outside	immediate	temporal	placing a silastic sheet over the condylar head	temporal bone graft	N	42	NR	NR	NR	4	32	to the L	NR
Nadal et al. (2005) [54]	NR	NR	open reduction with craniotomy	7	coronal and preauricular	NA	osteocartilaginous graft using the navicular bone	N	NR	NR	NR	NR	6	adequate	minimally to the L	NR
Cillo et al. (2005) [55]	NR	NR	open reduction with craniotomy with inferiorly directed force applied to the posterior mandible and inferior pressure placed on the condyle using a periosteal elevator	immediate	extended preauricular	repositioning the disk and placing a sheet of Gelfilm	calvarial bone graft	NR	21	NR	NR	NR	6	NR	to the R	NR
de Oliveira et al. (2005) [56]	NR	NR	open reduction, arthroplasty, and orthognathic surgery	1,825 (5 years)	modified preauricular	arthroplasty by interpositioning of an ear cartilage autogenous graft	ear cartilage autogenous graft among the osteotomized fragments	N	NR	Y	NR	orthodontic treatment and subsequent orthognathic surgery	NR	normal	NR	NR
Harstall et al. (2005) [57]	N	N	closed manual reduction	NR	closed	NA	NA	N	7	NR	NR	orthodontic treatment	36	35	4 mm to the R	shorter mandibular ramus on the R side
Soares et al. (2005) [58]	NR	NR	open manual reduction	NR	open	discectomy	temporalis muscle flap	NR	NR	Y	NR	NR	9	38	N	NR
Clauser et al. (2006) [59]	N	NR	open reduction with condylotomy	120	submandibular and preauricular	condylotomy by remodeling the bony surfaces using a rounded burr	temporalis muscle flap with inferior pedicle	N	14	Y	1	NR	1	45	NR	NR
Ohura et al. (2006) [60]	N	Y	L, open reduction with craniotomy by pushing the condyle downward; R, conservative	17	first, temporal; second, preauricular	open reduction by pushing the condyle downward	temporal fascial graft	redislocation on the fourth postoperative (first operation) day	21	NR	NR	N	24	42	NR	NR

Rosa et al. (2006) [61]	NA	NA	NA	NA	NA	NA	NA	N	NA	NA	NA	NR	NR	NR	NR	NR
Magge et al. (2007) [62]	NR	NR	open reduction with craniotomy	1	temporal	reduction externally with traction	split-thickness skull graft	N	28	Y	NR	NR	12	normal	slightly to the R	NR
Healy et al. (2008) [63]	N	Y	closed manual reduction ?	5	NA	NA	NA	N	42	Y	12	N	12	35	minimally to the L	NR
Taglialatela Scafati et al. (2008) [64]	N	N	open reduction with an anchor screw and condylectomy	21	preauricular	condylectomy with removal of few remnants of the disrupted disk	autogenous bone graft using bone chips and temporal fascia	NR	NR	NR	NR	NR	36	good function	N	NR
Menon & Sinha (2008) [65]	Y	Y	open reduction with condylotomy	7	preauricular	condylotomy with repositioning the remnants of the disk	ear cartilage autogenous graft among the osteotomized fragments	NR	7	NR	NR	NR	6	NR	to the L	NR
Lloyd & Sivarajasingam (2010) [66]	NR	Y	open reduction with craniotomy	immediate	preauricular and temporal	NA	NA	supraglottic edema	7	Y	1	NR	12	30	slightly to the L	NR
Man et al. (2011) [67]	NR	Y	open reduction by traction using Kocher forceps	10	submandibular and preauricular	disk repositioning	temporalis musculofascial flap and titanium network	NR	14	NR	NR	NR	12	restored	NR	NR
Hayashi et al. (2011) [68]	NR	NR	open reduction and reconstruction using a TMJ custom prosthesis	NR	retromandibular and endaural	reconstruction using a TMJ custom prosthesis	reconstruction using a TMJ custom prosthesis	NR	NR	NR	NR	NR	NR	NR	NR	NR
Struewer et al. (2012) [69]	N	NR	open reduction with front-temporal craniotomy, evacuation of the hematoma, osteosynthesis of the mandibular body and ramus and orbital floor	immediate	temporal	NA	temporalis muscular flap	N	N	NR	NR	NR	6	normal	NR	NR
Yoshida & Hyo (2012) [70]	N	Y	closed manual reduction	3	closed	NA	NA	N	N	Y	1	NR	32	28	to the L	NR
Jiao et al. (2013) [71]	Y	N	elastic intermaxillary traction and fixation	3	closed	NA	NA	N	7	Y	NR	N	24	35	N	NR
Garcia-Guevara et al. (2013) [72]	N	N	reconstruction using a temporomandibular joint prosthesis after waiting for the bone segment consolidation	210	preauricular and submandibular	arthrotomy cut at the level of the sigmoid notch	reconstruction using a temporomandibular joint prosthesis (Biomet/Lorenz Microfixation TMJ Replacement System)	N	N	Y	3	N	24	35	NR	NR
Lee et al. (2013) [73]	N	N	open reduction with craniotomy, partial mandibulectomy, and condylectomy	10	temporal, gingival-buccal, and transoral	partial mandibulectomy and condylectomy	deferred	N	NR	NR	NR	N	4	without trismus	NR	NR
Tutela et al. (2013) [74]	N	Y	closed manual reduction and external fixation	immediate	NA	NA	N	redislocation	16	NR	NR	N	1.5	37	NR	NR
Zhang et al. (2014) [75]	N	NR	open manual reduction ?	NR	semicoronal	repositioning and repair of the disk using a calvarial periosteum	repair using calvarial bone	N	NR	NR	NR	NR	12	41	NR	NR
	N	NR	open manual reduction ?	NR	preauricular	closed reduction of the R condyle	temporal musculofascial flap and titanium mesh	N	NR	NR	NR	NR	12	38	NR	NR
Vaezi et al. (2014) [76]	N	Y	open reduction with craniotomy using a periosteal elevator	NR	preauricular	disk repositioning	round-shaped miniplate	N	21	NR	NR	N	6	no complaint	slightly to the R	NR
Oberman et al. (2014) [77]	N	Y	closed manual reduction	NR	NA	NA	NA	N	7	NR	NR	NR	37 days	NR	NR	NR
Asai et al. (2014) [78]	N	Y	closed manual reduction	2	Closed	NA	NA	N	Y	Y	NR	N	8	38	5 mm to the R	NR
He et al. (2015) [79]	Y	Y	closed manual reduction	5	NA	NA	NA	N	30	Y	NR	orthodontic treatment	12	38	to the R	NR

	N	NR	open reduction with craniotomy	14	preauricular and coronal	reduction with intermaxillary screw drilled into the condyle and temporalis muscle graft	temporal bone graft fixed using a titanium miniplate	N	30	NR	NR	N	12	36	to the R	NR
	N	N	open reduction with craniotomy using a towel clamp and osteosynthesis of the mandibular body	150	preauricular and coronal	reduction using a towel clamp	temporal bone graft fixed using titanium plate	N	30	NR	NR	N	12	35	to the R	NR
Temiz et al. (2015) [80]	N	Y	closed manual reduction	within 16 hours	NA	NA	NA	N	14	Y	NR	N	6	32	N	N
	N	Y	open manual reduction	21	preauricular	removal of fibrotic tissues and callus	NA	N	Y	Y	NR	N	12	35	to the R	N
Zhang et al. (2016) [81]	N	NR	open reduction using a Kocher clamp	1	gingival labial and preauricular	incision of the joint capsule to confirm the position of the condyle	placement of Duragen to repair the skull base defect	N	Y	NR	NR	N	NR	NR	NR	NR
Arya & Chigurupati (2016) [82]	NR	Y	closed manual reduction	NR	NA	NA	NA	NR	Y	NR	NR	NR	NR	NR	NR	NR
	NR	Y	closed manual reduction	NR	NA	NA	NA	NR	NR	NR	NR	NR	NR	NR	NR	morphologic changes of the L condylar head
Kanno et al. (2016) [83]	N	N	open reduction with craniotomy by extraoral mandibular traction	3	coronal	removal of small fragments around the fractured roof of the glenoid fossa	cranial base reconstruction using a round-shaped miniplate	N	28	NR	NR	N	18	35	NR	N
Zamorano et al. (2016) [84]	N	N	open reduction with craniotomy, R condylectomy, and bilateral coronoidectomy	1,095	temporal, preauricular, and intraoral	R condylectomy, bilateral coronoidectomy	temporalis muscle flap	NR	NR	Y	NR	necessary orthodontic and surgical treatment	12	40	N	micrognathia
Rikhotso & Bobat (2016) [85]	N	N	bilateral arthroplasty accompanied by total joint replacement	365	bilateral preauricular	arthroplasty cut at the level of the sigmoid notch for removal of the extracranial ankylosed fragment of the dislocated condyle	bilateral total joint replacement using a Biomet Microfixation TMJ stock replacement system	NR	NR	Y	3	NR	36	35	N	NR
Pinares & Urzúa (2016) [86]	N	NA	NA	NA	NA	NA	NA	N	NA	NR	NR	NR	NR	NR	NR	NR
Yadegari et al. (2016) [87]	N	Y	closed manual reduction	0	closed	NA	NA	redislocation	N	Y	4	NR	36	25	N	NR
Lindell & Thor (2017) [88]	N	Y	first, closed manual reduction, reduction of symphysis fracture; second, arthroplasty accompanied by total joint replacement	0	preauricular and retromandibular	resection of the condylar head and coronoid process with custom-made cutting guide	L total joint replacement using a custom-made alloplastic TMJ prosthesis (Biomet Microfixation)	NR	14	Y	NR	NR	84	44	NR	NR
De Mol et al. (2017) [89]	NA	NA	conservative	NA	NA	NA	NA	N	NA	NA	NA	N	NR	NR	NR	NR
Liu et al. (2017) [90]	Y	Y	open reduction with gap arthroplasty	35	submandibular and preauricular	L gap arthroplasty and osteotomy between the neck of the L condyle and the sigmoid notch	temporalis fascial flap	NR	15	NR	NR	N	6	30	to the L	NR
Kurimori et al. (2018) [91]	N	N	open reduction with craniotomy and internal fixation of the L condylar and parasymphysis	8	temporal, preauricular, and submandibular	open reduction using a steel wire	temporalis muscle flap	NR	14	NR	NR	orthodontic treatment	36	NR	NR	L TMJ pain and click
	N	N	open reduction with gap arthroplasty and coronoidotomy	2,190	preauricular	subcondylar osteotomy and interposing a Silastic sheet	NA	N	NR	NR	NR	NR	36	acceptable	NR	NR

Monteiro et al. (2019) [92]	N	N	open manual reduction with craniotomy	20	temporal	removal of the bone in the medial and anterior aspect to the condyle using a Kerrison rongeur	temporal bone graft fixed using short titanium screws	vomiting, intense headache, and extravasation of the CSF through the R external auditory canal	Y	NR	NR	NR	5	22	NR	a discrete peripheral facial nerve deficit and diminished hearing on the R side
Liau et al. (2019) [93]	N	N	open reduction with traction	NR	preauricular	disk repositioning	the temporal bone fragment located and repositioned under endoscopic guidance	N	NR	Y	NR	NR	1.5	normal	NR	NR
Holz et al. (2019) [94]	NA	NA	NA	NA	NA	NA	NA	NA	NA	NA	NA	NA	NA	NA	NA	died after arrival at hospital
	NA	NA	NA	NA	NA	NA	NA	NA	NA	NA	NA	NA	NA	NA	NA	found in corpse
Chen et al. (2019) [95]	N	Y	open reduction with R condylectomy and coronoidectomy	17	intraoral	osteotomy at the condylar neck	NA	N	7	Y	3	NR	96	50	to the R	NR
Esquenazi et al. (2019) [96]	N	N	open reduction with craniotomy	0	preauricular and hemicoronal	open reduction	temporal musculofascial flap repaired using titanium mesh	N	Y	NR	NR	NR	NR	NR	NR	NR
Kyrpa et al. (2019) [97]	NR	NR	open reduction with craniotomy	2	temporal	removal of temporal bone fragments	temporal bone graft	N	NR	NR	NR	NR	NR	NR	NR	NR
Romano et al. (2019) [98]	N	NR	open reduction with craniotomy and fixation of multiple skull base fracture	0	NR	open reduction	temporalis muscle flap	hearing deficit associated with facial nerve paralysis	NR	NR	NR	N	12	NR	slight	NR
Congiusta & Champion (2020) [99]	NA	NA	closed manual reduction	NR	NA	NA	NA	NR	Y	NR	NR	NR	75	42	N	marked erosive bony changes, decreased joint space, and persistent dehiscence of the bony glenoid fossa
Ramani et al. (2021) [100]	NA	NA	closed manual reduction	NA	NA	NA	NA	NR	14	Y	2	N	120	25	N	remodeling with thick sclerotic bone in the lateral and posterior aspect of the L condyle, anterior disk displacement without reduction in the R TMJ
Díez-Suárez & Paredes-Farrera (2021) [101]	Y	Y	open reduction with craniotomy and condylotomy	7	preauricular and temporal	removal of the unstable segment of the diacapitular fracture and condylar remodeling	reconstruction using a resorbable mesh and temporalis muscle flap	NR	NR	Y	14	orthodontic treatment	12	40	to the L	persistent mandibular deviation
Zumbrunn Wojczyńska et al. (2021) [102]	NA	NA	conservative	NA	NA	NA	NA	N	NA	NA	NA	N	12	no progression	NR	NR
Motazedian et al. (2021) [103]	NA	NA	conservative	NA	NA	NA	NA	N	NA	NA	NA	N	NR	NR	NR	NR
D'Hondt et al. (2022) [104]	NA	NR	subcondylar osteotomy and mandibular debridement	90	NR	mandibular debridement	temporal fascia flap	development of the L facial nerve paresis	NR	NR	NR	N	12	30	to the L	NR

Y, yes; N, no; NR, not reported; NA, not applicable; L, left; R, right; TMJ, temporomandibular joint; CSK, cerebrospinal fluid.
